# Publisher Correction: Live-cell three-dimensional single-molecule tracking reveals modulation of enhancer dynamics by NuRD

**DOI:** 10.1038/s41594-023-01179-1

**Published:** 2023-12-15

**Authors:** S. Basu, O. Shukron, D. Hall, P. Parutto, A. Ponjavic, D. Shah, W. Boucher, D. Lando, W. Zhang, N. Reynolds, L. H. Sober, A. Jartseva, R. Ragheb, X. Ma, J. Cramard, R. Floyd, J. Balmer, T. A. Drury, A. R. Carr, L.-M. Needham, A. Aubert, G. Communie, K. Gor, M. Steindel, L. Morey, E. Blanco, T. Bartke, L. Di Croce, I. Berger, C. Schaffitzel, S. F. Lee, T. J. Stevens, D. Klenerman, B. D. Hendrich, D. Holcman, E. D. Laue

**Affiliations:** 1 Department of Biochemistry, University of Cambridge, Cambridge, UK; 2 Wellcome-MRC Cambridge Stem Cell Institute, Jeffrey Cheah Biomedical Centre, Cambridge, UK; 3 Department of Physiology, Development and Neuroscience, University of Cambridge, Cambridge, UK; 4 Department of Applied Mathematics and Computational Biology, Ecole Normale Supérieure, Paris, France; 5 Yusuf Hamied Department of Chemistry, University of Cambridge, Cambridge, UK; 6 The European Molecular Biology Laboratory EMBL, Grenoble, France; 7 Centre for Genomic Regulation (CRG), The Barcelona Institute of Science and Technology, Barcelona, Spain; 8 Helmholtz Zentrum München, German Research Center for Environmental Health, Institute of Functional Epigenetics, Neuherberg, Germany; 9 Institució Catalana de Recerca i Estudis Avançats (ICREA), Barcelona, Spain; 10 School of Biochemistry, University of Bristol, Bristol, UK; 11 MRC Laboratory of Molecular Biology, Cambridge Biomedical Campus, Cambridge, UK; 12 Present Address: School of Physics and Astronomy, University of Leeds, Leeds, UK; 13 Present Address: School of Food Science and Nutrition, University of Leeds, Leeds, UK; 14 Present Address: Centre for Biodiversity Genomics, University of Guelph, Guelph, Ontario Canada; 15 Present Address: The European Molecular Biology Laboratory, Heidelberg, Germany; 16 Present Address: Sylvester Comprehensive Cancer Center, Department of Human Genetics, University of Miami Miller School of Medicine, Biomedical Research Building, Miami, FL USA

**Keywords:** Chromatin structure, Chromatin remodelling, Pluripotent stem cells, Cellular imaging

Correction to: *Nature Structural & Molecular Biology* 10.1038/s41594-023-01095-4, published online 28 September 2023.

In the version of the article initially published there were some errors in the affiliations. A. Ponjavic’s second and third affiliations have been corrected to Present address: School of Physics and Astronomy, University of Leeds, Leeds, UK and Present address: School of Food Science and Nutrition, University of Leeds, Leeds, UK; and L. Morey now has only two affiliations: Centre for Genomic Regulation (CRG), The Barcelona Institute of Science and Technology, Barcelona, Spain and Present address: Sylvester Comprehensive Cancer Center, Department of Human Genetics, University of Miami Miller School of Medicine, Biomedical Research Building, Miami, FL, USA. Additionally, the received date has been corrected to 14 April 2020 from 26 October 2021. These errors have been corrected in the HTML and PDF versions of the article.

