# Supplementary material for: Live-cell three-dimensional single-molecule tracking reveals modulation of enhancer dynamics by NuRD
Source: Nat Struct Mol Biol. 2023 Sep 28;30(11):1628–39. doi: 10.1038/s41594-023-01095-4 (PMC10643137; doi:10.1038/s41594-023-01095-4)
Supplement: Supplementary file 1 — Supplementary Data Fig. 1, Tables 1–7, video list and Methods. [file 41594_2023_1095_MOESM1_ESM.pdf]

nature structural & molecular biology

Article https://doi.org/10.1038/s41594-023-01095-4

Live-cell three-dimensional single-molecule tracking reveals modulation of enhancer dynamics by NuRD

In the format provided by the authors and unedited

Table of Contents

Supplementary Data Figures 2
Supplementary Figure 1. 2
Supplementary Data Tables 2
Supplementary Table 1 2
Supplementary Table 2 3
Supplementary Table 3 3
Supplementary Table 4 3
Supplementary Table 5. 3
Supplementary Table 6 4
Supplementary Table 7 4
Supplementary Data Videos 4
Supplementary Methods 5

Supplementary Data Figures

Supplementary Figure 1.

Evaluating the classifier using ground-truth trajectories with known classes (confined C and unconfined U). (a) The switching behaviour is described in Eq. 28, see below, where the class (C, U) is determined at each time point using a Markov chain (left) with switching rates λ, μ for the transitions between C and U and vice-versa, respectively (right). (b) After the ground truth set of trajectories was generated, we applied the classification procedure as described below, and computed the accuracy measure M (Eq. 35) defined as the fraction of correctly assigned classes by the segmentation algorithm according to the known classes in the ground-truth set. (Left) We computed M for trajectories generated using Eq. 29 with diffusion coefficients $D_c \in [0.002,0.1]$ $\mu m^2/s$ for class C and $D_U \in [0.2,1]$ $\mu m^2/s$ for U. We find that $M > 0.76$ for all tested values of D_C, D_U, while a well separated D_C, D_U resulted in $M > 0.9$. (Right) The accuracy M for switching rates λ, $\mu \in [0,1]$ and $D_C = 0.008$, $D_U = 0.01 \mu m^2/s$, M, resulted in $M > 0.85$ for all tested λ, μ.

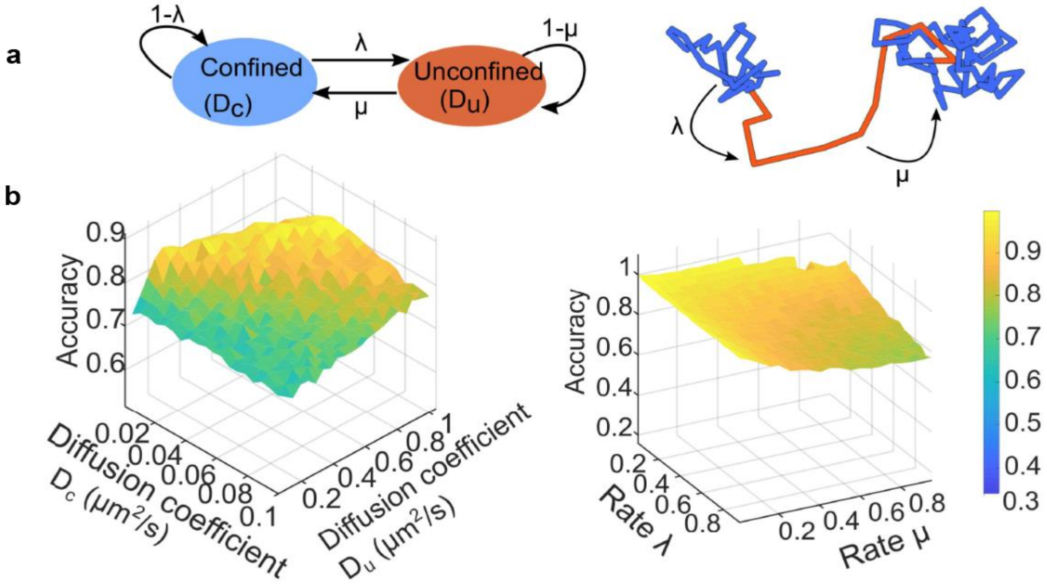

Supplementary Data Tables

Supplementary Table 1

***In situ* Hi-C**			
**Experiment**	**Source**	**Condition**	**Unique Contacts**
ERR2391437	E-MTAB-6591	WT	131,434,520
ERR2391439	E-MTAB-6591	WT	133,825,748
ERR2391441	E-MTAB-6591	WT	144,255,897
ERR2391443	E-MTAB-6591	WT	132,181,206
SLX-7672	This study	WT	55,178,175
SLX-7676	This study	Mbd3-ko	64,883,460
SLX-18035	This study	Mbd3-ko	124,812,397
SLX-19611_s_1	This study	Mbd3-ko	69,008,264
SLX-19611_s_2	This study	Mbd3-ko	68,483,385

Supplementary Table 2

ANTIBODY	RAISED IN	COMPANY	CATALOGUE NUMBER	WESTERN BLOT	ChIP	Clonality
αCTCF	Rabbit	Millipore	07-729	1:1000	5μl	Polyclonal
αSMC3	Rabbit	Abcam	ab9263	1:1000	5μl (5μg)	Polyclonal

Supplementary Table 3

ANTIBODY	RAISED IN	COMPANY	CATALOGUE NUMBER	WESTERN BLOT	Clonality
αCHD4	Mouse	Abcam	ab70469	1:5000	Monoclonal [3F2/4]
αFlag	Mouse	Sigma	F1804	1:5000	Monoclonal M2
αGATAD2A	Rabbit	Abcam	ab87663	1:2000	Polyclonal
αHDAC1	Rabbit	Abcam	ab7028	1:2000	Polyclonal
αMBD3	Rabbit	Abcam	ab157464	1:5000	Monoclonal [EPR9913]
αMTA2	Mouse	Abcam	ab50209	1:5000	Monoclonal [MTA2-276]
αPCNA	Mouse	Santa cruz	Sc56	1:2000	Monoclonal [PC10]

Supplementary Table 4

Parameter estimation from trajectories with Δt = 20 ms.

	D	BIC	K_c	K_u	D_c	D_u	BIC	K_c	K_{u1}	K_{u2}	D_c	D_{u1}	D_{u2}	BIC	Ndisps
**CHD4**	0.206	-1718	0.58	0.42	0.118	0.616	-2932	0.23	0.56	0.21	0.068	0.222	1.126	-3268	237358
**CHD4-MBD3**	0.234	-1707	0.54	0.46	0.121	0.649	-2971	0.29	0.48	0.23	0.085	0.271	1.134	-3093	85046
**CHD4-GATA**	0.17	-1750	0.51	0.49	0.088	0.382	-2986	0.26	0.57	0.17	0.061	0.199	0.856	-3158	138121
**MBD3**	0.147	-1743	0.51	0.49	0.076	0.319	-2966	0.28	0.59	0.13	0.054	0.181	0.813	-3096	75809
**MTA2**	0.194	-1786	0.45	0.55	0.096	0.384	-2866	0.16	0.62	0.22	0.056	0.192	0.715	-2794	13767
**MTA2-MBD3**	0.383	-1628	0.5	0.5	0.174	1.2	-2805	0.16	0.46	0.38	0.065	0.279	1.495	-2908	24283

Supplementary Table 5.

Parameter estimation from trajectories with Δt = 500 ms. In both Tables 1 and 2, D_c and D_u are the estimated apparent diffusion coefficients (whilst K_c and K_u are their relative proportions). The blue boxes indicate our preferred model based on BIC analysis.

	D	BIC	K_c	K_u	D_c	D_u	BIC	K_c	K_{u1}	K_{u2}	D_c	D_{u1}	D_{u2}	BIC	Ndisps
**CHD4**	0.005	-1874	0.55	0.45	0.003	0.01	-3089	0.16	0.68	0.16	0.002	0.005	0.017	-3170	91470
**CHD4+DRB**	0.003	-1796	0.46	0.54	0.002	0.006	-3036	0.24	0.63	0.13	0.001	0.004	0.014	-3062	34164
**CHD4-MBD3**	0.004	-1855	0.52	0.48	0.002	0.008	-3145	0.2	0.63	0.17	0.002	0.004	0.014	-3218	60964
**MBD3**	0.004	-1827	0.55	0.45	0.003	0.009	-3094	0.22	0.62	0.16	0.002	0.005	0.017	-3205	53176
**MBD3+FK228**	0.004	-1803	0.56	0.44	0.002	0.008	-3151	0.36	0.51	0.13	0.002	0.005	0.017	-3181	64328

Supplementary Table 6

ID	frame	x	y	z	confinementScore	confined	alpha	beta	diffusion	Lc	Kc	driftMagnitude
6	1778	27361	28318	-1700.2	7.29E-59	0	0.571	0.060	0.048	129.860	0.594	0.120
6	1779	27723	28205	-1648.9	2.41E-42	0	0.544	0.050	0.040	119.747	0.479	0.060
6	1780	27388	28286	-1383.2	1.48E-37	0	0.665	0.075	0.042	132.412	0.396	0.114
6	1781	27598	28117	-1419.6	3.65E-36	0	0.620	0.063	0.040	125.842	0.309	0.077
6	1782	27394	28360	-1747.8	1.14E-30	0	0.595	0.061	0.039	127.778	0.279	0.086
6	1783	27619	28289	-1548.6	1.27E-29	0	0.529	0.051	0.037	122.881	0.236	0.037
6	1784	27513	28323	-1602.4	1.13E-15	0	0.573	0.051	0.032	119.783	0.228	0.067
6	1785	27265	28367	-1316	5.54E-11	0	0.601	0.049	0.029	117.339	0.241	0.056
6	1786	27511	28317	-1431.7	2.19E-10	0	0.620	0.052	0.029	118.620	0.279	0.088
6	1787	27733	28230	-1649	1.81E-05	0	0.574	0.040	0.024	109.199	0.265	0.035
6	1788	27539	28282	-1666.7	0.002652771	0	0.625	0.041	0.022	107.263	0.283	0.075
6	1789	27412	28329	-1631.1	1.48E-08	0	0.627	0.046	0.026	114.530	0.334	0.042

Supplementary Table 7

**Locus**	**BAC library code**	**Probe label**
Tbx3 promoter	BMQ-338M19	Cy3
Tbx3 enhancer	BMQ-309K22	Alexa Fluor 647
Sox2 promoter	BMQ-158G1	Cy3, Alexa Fluor 647
Sox2 enhancer	BMQ-53J20	Alexa Fluor 647
Bmp4 promoter	BMQ-116K2	Cy3
Bmp4 enhancer	BMQ-249B4	Alexa Fluor 647

Supplementary Data Videos

Supplementary Video 1: CHD4_20ms.avi

Supplementary Video 2: CHD4_500ms.avi

Supplementary Video 3: CHD4_20ms_Cell1.avi

Supplementary Video 4: CHD4_20ms_Cell2.avi

Supplementary Video 5: CHD4_20ms_Cell3.avi

Supplementary Video 6: CHD4_500ms_Cell1.avi

Supplementary Video 7: CHD4_500ms_Cell2.avi

Supplementary Video 8: CHD4_500ms_Cell3.avi

Supplementary Video 9: Nanog_enhancer.avi

Supplementary Video 10: Tbx3_enhancer.avi

Supplementary Video 11: Nanog_enhancer_tracks.avi

Supplementary Video 12: Tbx3_enhancer_tracks.avi

(In the videos showing the tracking of Nanog/Tbx3 enhancer loci (Supplementary Videos 11 and 12) the gradient in the colours of the tracks represents the motion over time, with purple at the start, through green to yellow at the end of the video.)

Supplementary Methods

Single-molecule trajectory analysis

We describe below (see Section 1) a switching model for a stochastic process between two or more states, characterized by different diffusion coefficients. We initially used this approach to decide whether we should develop a two- or three-state diffusion model to classify the distribution of displacements measured along single molecule trajectories. We then went on to develop an algorithm to classify sub-trajectories into confined and unconfined states based on four physical parameters using a Gaussian mixture model. This is described in Section 2. The algorithm was then tested on simulated trajectories which provided ground truth data. Finally, in Section 3, we describe how we applied this classification algorithm to single particle trajectories to estimate the diffusion coefficients in confined and unconfined states. However, the original classification based on diffusion coefficients provided a complementary analysis, and justified the results we obtained later, which is why it is included here.

1. Switching dynamics and displacement analysis

We describe the motion of a particle that switches between different states characterized by different diffusion coefficients[89,90]. We initially used this description to identify the most appropriate number of switching states, based on the distribution of the length of the instantaneous displacements $\Delta X = X(t+\Delta t)-X(t)$. First, we describe the switching dynamics and fitting procedure, and then how we used the Bayesian Information Criterion (BIC) to select the optimal model.

1.1 Stochastic motion described by a diffusion model

The dynamics of a chromatin-binding protein complex such as NuRD, or some component of it, can be described by Langevin's equation[91,92] in the classical overdamped limit, where the position $X(t)$ satisfies:

$$\dot{\boldsymbol{X}} = \frac{\boldsymbol{f}(\boldsymbol{X})}{\gamma} + \sqrt{2D}\dot{\boldsymbol{\eta}}, \qquad (1)$$

where the force f depends on the position X and the second term corresponds to steady-state diffusion in a crowded medium, characterized by a constant diffusion coefficient D. Here η is a Gaussian variable with mean 0 and variance 1. When the protein binds to its molecular partners or chromatin, the diffusion coefficient could change, and the motion can become restricted, e.g. to that along the path of the DNA.

Experimental trajectories consist of a series of points ($X(k\Delta t)$ k = 1... and in the absence of any additional localization error noise, or an external force f = 0, the displacement dynamics at the sampling rate interval Δt, is given by:

$$\boldsymbol{X}(t+\Delta t) = \boldsymbol{X}(t) + \sqrt{2D\Delta t}\boldsymbol{\eta}, \qquad (2)$$

where η is a vector of Gaussian values of mean 0 and variance 1. In two dimensions, the distribution of displacements is:

$$Pr\{|\Delta\boldsymbol{X}| = u\} = \frac{u}{\sigma^2}\exp\left(-\frac{u^2}{2\sigma^2}\right), \qquad (3)$$

where $\sigma^2 = 2D\Delta t$ (see Eq. 5). For empirical displacements that contain a Gaussian localization error, characterized at the time resolution Δt by the amplitude σ_{le}, the formula should be modified[93]:

$$Pr\{|\Delta\boldsymbol{X}| = u\} = \frac{u}{2D\Delta t + \sigma_{le}}\exp\left(-\frac{u^2}{2(2D\Delta t + \sigma_{le}^2)}\right) \qquad (4),$$

We can thus use the approximated diffusion coefficient:

$$D_{app} = D + \frac{\sigma_{le}^2}{2\Delta t}. \qquad (5)$$

1.2 Modelling stochastic switching behaviour

We next constructed a model for a chromatin-binding protein by using a classical Markov chain with two states *1* and *2*, characterized by rate constants λ and µ for switching between the states, and by the diffusion coefficients D_1 and D_2. The associated jump process is defined by:

$$X(t+\Delta t) = X(t) + \begin{cases} \sqrt{2D_1\Delta t}\eta & \text{w.p.} 1-\lambda\Delta t \\ 0 & \text{w.p.} \lambda\Delta t \end{cases}, \tag{6}$$

$$X(t+\Delta t) = X(t) + \begin{cases} \sqrt{2D_2\Delta t}\eta & \text{w.p.} 1-\mu\Delta t \\ 0 & \text{w.p.} \mu\Delta t \end{cases}, \tag{7}$$

where the transition rates from states 1 to 2 are described by the switching equation:

$$1 \underset{\lambda}{\overset{\mu}{\rightleftharpoons}} 2.$$

The probability density function (pdf) p_d for the displacement of a molecule depends on the state of the process and can be computed using Bayes' law:

$$Pr_d\{|\Delta X| = u\} = Pr\{|\Delta X| = u|X(t) \text{ and } X(t+\Delta t) \text{in state } 1\}q_1(t)$$

$$+Pr\{|\Delta X| = u|X(t) \text{ and } X(t+\Delta t) \text{ in state } 2\}q_2(t), \tag{8}$$

where using Bayes relation, for state $i = 1$ or 2,

$$q_i(t) = Pr\{X(t) \text{ and } X(t+\Delta t) \text{ in state i}\} = Pr\{X(t+\Delta t) \text{in state i}|X(t) \text{ in state i}\}$$

$$\times Pr\{X(t) \text{ in state i}\}.$$

Because $p_i(t) = Pr\{X(t) \text{ in state i}\}$ is the solution to the Master equation:

$$\frac{d}{dt}p_1 = -\lambda p_1 + \mu p_2 \tag{9}$$

$$\frac{d}{dt}p_2 = \lambda p_1 - \mu p_2. \tag{10}$$

and

$$\begin{aligned} p_1(t) &= \kappa + Ce^{-(\lambda+\mu)t} \\ p_2(t) &= 1 - \kappa - Ce^{-(\lambda+\mu)t}, \end{aligned}$$

where $\kappa = \frac{\lambda}{\lambda+\mu}$ and C depend on the initial distribution, we have:

$$Pr\{X(t+\Delta t) \text{in state } 1|X(t) \text{ in state } 1\} = 1 - \lambda\Delta t, \text{ and}$$

$$Pr\{X(t+\Delta t) \text{in state } 2|X(t) \text{ in state } 2\} = 1 - \mu\Delta t.$$

We conclude that in two-dimensions, for a long-time t and a short-time Δt, using Eqs. 8 and 3, the pdf can be written as:

$$Pr_d\{|\Delta\boldsymbol{X}| = u\} = \kappa\frac{u}{\sigma_1^2}\exp\left(-\frac{u^2}{2\sigma_1^2}\right) + (1-\kappa)\frac{u}{\sigma_2^2}\exp\left(-\frac{u^2}{2\sigma_2^2}\right) \quad (11),$$

where $\sigma_k = 2D_k\Delta t$ and $k = 1,2$, and κ is the ratio of the time spent in a confined state. In the case of a three-state model, the pdf for the displacement of a molecule is:

$$\begin{aligned} Pr_{\text{switch}}\{\Delta\boldsymbol{X} = u\} &= \kappa_1\frac{u}{\sigma_1^2}\exp\left(-\frac{u^2}{2\sigma_1^2}\right) + \kappa_2\frac{u}{\sigma_2^2}\exp\left(-\frac{u^2}{2\sigma_2^2}\right) \\ &+(1-\kappa_1-\kappa_2)\frac{u}{\sigma_3^2}\exp\left(-\frac{u^2}{2\sigma_3^2}\right), \end{aligned} \quad (12)$$

where $\sigma_k = 2D_k\Delta t$ with k = 1, 2, and 3 and D_1, D_2, and D_3 are the three diffusion coefficients. Here κ_1 (respectively κ_2) is the fraction of time spent in state 1 (respectively state 2).

To test the appropriateness of a one-, two- or three-state models, we extracted the diffusion coefficients from Eqs. 11-12 and the associated rate constants. We then estimated the quality of fit of the different models by using the Bayesian Information Criterion (BIC) defined as:

$$BIC = (p+1)n(\ln n)\left[\ln\left(\frac{2\pi\text{RSS}}{n}\right)+1\right] \quad (13)$$

where p is the number of parameters of the model from Eq. 12, n is the number of data points, and RSS is the residual sum of squares between the model and the data.

Analysis of the 20 ms trajectories revealed that the data could be characterized by either a two- or a three-state model, consisting of a confined and either one or two unconfined states (see **Supplementary Table 4**). Similarly, we evaluated 1, 2 and 3 state models for analysis of the 500 ms data consisting of a confined and either one or two sub-diffusive states (see **Supplementary Table 5**). Although this showed that a three-state model could best describe both the 20 and 500 ms data, the analysis also revealed that the BIC values for the three-state model were only slightly larger, suggesting that a two-state model would be sufficient to explain the data.

In summary, the displacement analysis revealed that a two-state diffusion model should be appropriate to analyse NuRD complex dynamics: in both cases there was only a small improvement in the BIC for the three-state diffusion model, suggesting that the 20 ms trajectories can be characterised by confined (low diffusion coefficient D) and unconfined (high diffusion coefficient D) states, whilst the 500 ms data can be characterised by slow and fast sub-diffusive chromatin bound states. However, this analysis supported our later identification of a slow and two fast states in the 500 ms data (see Section 5, below).

2. Gaussian mixture model for classification and segmentation of trajectories into two states using four biophysical parameters (4P)

The Gaussian classification described in Section 1 above, is limited as it considers each displacement as being independent and then pools them, destroying any possible causality present in the trajectories. Moreover, because parts of our trajectories could correspond to molecules that are confined (i.e. chromatin bound) and other parts to when they are unconfined (or diffusing more freely), our trajectories exhibit a variety of behaviours characterised not only by slow and fast diffusion coefficients but also by smaller/larger anomalous exponents, the exploration of smaller/larger regions of space, and less/more directional movement. We therefore needed an algorithm that took into account these types of behaviour and not just changes in diffusion coefficient. Based on recent work to characterise such biophysical parameters[40], we present here an algorithm that uses these parameters to classify and segment a trajectory into two states – when analysing 20 ms data the resulting sub-trajectories are confined (C) and unconfined (U) in the associated four dimensional space, whilst for the 500 ms data, we observe two confined states, slow- and fast-diffusing (see **Figure 1** in the main text). The method is based on a generalized Gaussian mixture model[94], where the input data is an ensemble of trajectories and the output is two ensembles of sub-trajectories.

2.1 Input data

We obtain an ensemble of N trajectories $X_i(k\Delta t)$, $i = 1,...N$, $k = 0,1,...,n_i$, with an acquisition time step Δt, such that each trajectory consists of n_i discrete points in three dimensions.

2.2 Statistical features are extracted in a sliding window along the trajectories

To classify sub-trajectories as confined C or unconfined U, we used four physical parameters[38,95], computed along single trajectories. For a trajectory given by the successive points $X_i(k\Delta t)$, we used a sliding window W_k containing $2l + 1$ points, centered at $X(k\Delta t)$, and defined as:

$$W_k^{(d)}(m\Delta t) = \{X_i^{(d)}((k-l+m)\Delta t), m = 0, \ldots, 2l, \quad d = 1, 2 \text{ and } 3\}, \tag{14}$$

where d is the space dimension. The sliding window $W_k(m\Delta t)$ is applied for $k = 1,\ldots n_i$ along a trajectory containing n_i points.

We now briefly discuss the four parameters we used for the classification based on previous work which showed that they can be used individually for the classification of single-molecule trajectories:

Anomalous exponent α: The anomalous exponent characterizes the motion of a stochastic particle on a particular time scale involving several time steps Δt. It is computed from the Mean-Square-Displacement (MSD) $\langle|X(t+\Delta t) - X(t)|^2\rangle$ that behaves like Δt^{α} where Δt is small compared to the time of the process. A value of $\alpha = 1$ reflects Brownian motion, and an $\alpha > 1$ is called super-diffusion, which may represent dynamics containing an element of deterministic (ballistic) directed motion. Finally, an $\alpha < 1$ is sub-diffusive motion and has been used previously to study changes in chromatin compaction[38,96,97]. To estimate α for each point X, we first compute the MSD S_k over the sliding window $W_k(m\Delta t)$ classically defined by:

$$S_k(m) = \langle\|W_k(m\Delta t) - W_k(0))\|^2\rangle, m = 0, \ldots, 2l, \tag{15}$$

Where $\langle\cdot\rangle$ denotes the average where we use the intermediate point from 1 to m. To estimate the exponent $\alpha_i(k)$ for point $X(k\Delta t)$, we fitted the function $S_k(m)$ computed by summing over each displacement contributing to Eq. (15) with:

$$f_k(t) = \beta_i(k)t^{\alpha_i(k)} \tag{16}$$

where $\beta_i(k) > 0$. For the fit, we constrain the variable t in the ensemble $[0,\Delta t,\ldots,(2l+1)\Delta t]$.

Effective Diffusion coefficient D**:** We estimate the effective diffusion coefficient[95,98] by computing the second statistical moment along the trajectories. We use the empirical estimator to estimate for each sliding window $W_k(m\Delta t)$[38]:

$$D_i(k) = \frac{1}{2d\Delta t}\sum_{m=0}^{2l}(W_k((m+1)\Delta t)) - W_k(m\Delta t))^2 \quad . \tag{17}$$

Length of confinement *Lc*. The length of confinement estimates the size of a domain where a trajectory is confined. It is computed empirically in the window W_k by:

$$Lc_i(k) = \frac{1}{3}\sum_{d=1}^{3}\sqrt{\frac{1}{2l}\sum_{m=0}^{2l}(W_k^{(d)}(m\Delta t) - \langle W_k^{(d)}\rangle)^2} = STD(W_k), \tag{18}$$

where:

$$\langle W_k^{(d)}\rangle = \frac{1}{2l}\sum_{m=0}^{2l} W_k^{(d)}(m\Delta t), d = 1, 2 \text{ and } 3. \tag{19}$$

It is the standard deviation of the sub-trajectory W_k, where the average position is ($\langle W_k^{(1)}\rangle, \langle W_k^{(2)}\rangle, \langle W_k^{(3)}\rangle)$.

Magnitude of the drift vector V_i (k)**.** To characterize the displacement of a trajectory between the beginning and the end of the sliding window W_k, we compute the magnitude V_i (k) of the drift vector V_i for each dimension d = 1,2,3, [98] using the formula:

$$V_i^{(d)}(k) = \frac{1}{2l\Delta t}\sum_{m=0}^{2l-1} W_k^{(d)}((m+1)\Delta t) - W_k^{(d)}(m\Delta t) \quad . \tag{20}$$

The norm of the drift V_i (k) is:

$$\|V_i\| = \sqrt{\sum_{d=1}^{3}(V_i^{(d)}(k))^2.} \tag{21}$$

In summary, we compute four parameters: the anomalous exponent α, the apparent diffusion coefficient D, the length of confinement Lc, and the magnitude of the drift vector V_i (k). These are all computed for each point along a trajectory using a sliding window. The sliding window of 11 points was chosen by trial and error. Below this value, the anomalous exponent distributions for confined and unconfined molecules tended to merge suggesting that long trajectories are essential for reliable estimation of this parameter. Above this value, the diffusion coefficient histograms for confined and unconfined molecules tended to merge suggesting that transitions between these populations occur leading to averaging of the diffusion coefficients. In the next section, we use these four parameters for classification.

2.3 Classification of a discrete time point in a trajectory into either a confined (C) or an unconfined (U) state

To classify each time point $X((k-1)\Delta t)$ for $k = 1,...,n_i$ of all trajectories indexed by $i = 1,...,N$ into C and U classes, we first collected all the values from Eqs. 16-21, computed from each sliding window W_k (Eq. 14). This led to a total of $n_i \times 4$ parameters, that we organized in a matrix R_i associated with trajectory *i:*

$$R_i = [\alpha_i^T, D_i^T, Lc_i^T, \|V\|_i^T] = \begin{bmatrix} r_i(\Delta t) \\ r_i(2\Delta t) \\ . \\ . \\ r_i(n_i \Delta t) \end{bmatrix}, \tag{22}$$

where T is the transpose operator, and $r_i(k\Delta t)$ are four dimensional vectors of the parameters (Eqs. 16-18, 21). $\left(\sum_i^N n_i\right) \times 4$

We then concatenate all feature matrices R_i (Eq. 22), $i = 1,...N$ into the general matrices $\boldsymbol{R}$, defined by:

$$\boldsymbol{R} = \begin{bmatrix} R_1 \\ R_2 \\ . \\ . \\ R_N \end{bmatrix}. \tag{23}$$

and we normalize each column (feature) in $R^{(j)}, j = 1,...,4$ by subtracting its mean and dividing by its standard-deviation.

To separate the histograms of the four parameters into two independent classes, we constructed an unsupervised binary classifier using a two-component Gaussian mixture model in a four-dimensional space, corresponding to the four parameters Eqs. 16-21. The Gaussian mixture distribution p is a weighted sum of two multivariate Gaussian densities, defined as:

$$p(r|w_{C,U},\mu_{C,U},\Sigma_{C,U}) = w_C g(r|\mu_C,\Sigma_C) + w_U g(r|\mu_U,\Sigma_U), \qquad (24)$$

where w_C, w_U are the mixing weights, such that $w_C + w_U = 1$, and $g(r|\mu,\Sigma)$ are the Gaussian densities

$$g(r|\mu,\Sigma) = \frac{1}{(2\pi)^2|\Sigma|^{1/2}} \exp\Big(-\frac{1}{2}(r-\mu)^T\Sigma^{-1}(r-\mu)\Big), \qquad (25)$$

and Σ_C,Σ_U and μ_C,μ_U are the four-dimensional covariance matrices and mean vectors for components C and U, respectively. We then find the values of the parameters $\mu_C,\mu_U,\Sigma_C,\Sigma_U$ and w_C,w_U of Eq. 24 which best separate the data as the maximal likelihood estimators of the density (Eq. 25), given the observed statistics in R (Eq. 23), by using the Expectation-Maximization algorithm[99].

For each point $X_i(k\Delta t)$, with its associated feature vector $r_i(k\Delta t)$ (Eq. 23), we assign a label $n \in \{C,U\}$, based on the posterior probability P of the density Eq. 25, given by:

$$P(n|r_i(k\Delta t), w_n, \mu_n, \Sigma_n) = \frac{w_n g(r_i(k\Delta t)|\mu_n,\Sigma_n)}{w_{\mathcal{C}} g(r_i(k\Delta t)|\mu_{\mathcal{C}},\Sigma_{\mathcal{C}}) + w_{\mathcal{U}} g(r_i(k\Delta t)|\mu_{\mathcal{U}},\Sigma_{\mathcal{U}})} \qquad (26)$$

such that for each point:

$$X_i(k\Delta t) \in \begin{cases} \mathcal{C}, & Pr(\mathcal{C}|r_i(k\Delta t), w_{\mathcal{C}}, \mu_{\mathcal{C}}, \Sigma_{\mathcal{C}}) > Pr(\mathcal{U}|r_i(k\Delta t), w_{\mathcal{U}}, \mu_{\mathcal{U}}, \Sigma_{\mathcal{U}}) \\ \mathcal{U}, & \text{else.} \end{cases} \qquad (27)$$

yielding a segmented trajectory where each time point is assigned as confined (C) or unconfined (U). The resulting ensembles of sub-trajectories can then be analysed further, in particular to estimate the four biophysical parameters (Eqs. 16-18, 21). In practice, if a point $X_i(k\Delta t) \in$ C or U is isolated, e.g. C between two neighbouring U class points, then we relabel it as the class of its two immediate time neighbours $(k-1)\Delta t$ and $(k+1)\Delta t$.

3. Simulations to validate our 4P classification algorithm

In this section, we estimate the accuracy of the classification algorithm (subsections 2.2-2.3) using simulated (synthetic) trajectories that can switch between two states C and U (**Supplementary Figure 1a**). The dynamics were described by an Ornstein-Uhlenbeck equation[95] (Eq. 28), and the generated trajectories were considered as a ground-truth ensemble, which we used to estimate the accuracy of the classification procedure developed in the previous section.

3.1 Simulation of a ground-truth ensemble of synthetic trajectories

We generated N trajectories $X_i(t)$, $i = 1,\ldots,n_i$, which can switch between confined C and unconfined U states at any time t. The stochastic switching process is defined by:

$$\frac{dX(t)}{dt} = \begin{cases} -\kappa(X(t) - X(\tau)) + \sqrt{2D_{\mathcal{C}}}\frac{d\boldsymbol{\omega}}{dt}, & X(t) \in \mathcal{C}; \\ \sqrt{2D_{\mathcal{U}}}\frac{d\boldsymbol{\omega}}{dt}, & X(t) \in \mathcal{U}, \end{cases} \quad (28)$$

where D_C, and D_U are the diffusion coefficients for the confined C and unconfined U states respectively, $d\omega/dt$ are standard three-dimensional Brownian motions, with a mean of zero and a standard deviation of one, while κ is the strength of a potential well that attracts the trajectory $X(t) \in$ C to a fixed-point $X(\tau)$, which is the last position, before time t where the trajectory was in state U prior to switching to C:

$$\tau = \sup\{s < t, X(s) \in U \mid X(t) \in C\}. \quad (29)$$

The transition probability between states (C, U) is defined by a Markov chain: The transient probability matrix P_{ij} between state j at time $s + \Delta s$ and state i at time s, is given by:

$$P_{ij} = \begin{cases} \lambda\Delta s, & i = \mathcal{C}, j = \mathcal{U}; \\ 1 - \lambda\Delta s, & i = \mathcal{C}, j = \mathcal{C}; \\ \mu\Delta s, & i = \mathcal{U}, j = \mathcal{C}; \\ 1 - \mu\Delta s, & i = \mathcal{U}, j = \mathcal{U}. \end{cases} \quad (30)$$

The probabilities $P_C(s)$ and $(P_U(s))$ satisfy[100]:

$$\begin{bmatrix} \dot{P}_{\mathcal{C}}(s) \\ \dot{P}_{\mathcal{U}}(s) \end{bmatrix} = \begin{bmatrix} -\lambda & \mu \\ \lambda & -\mu \end{bmatrix} \begin{bmatrix} P_{\mathcal{C}}(s) \\ P_{\mathcal{U}}(s) \end{bmatrix}. \quad (31)$$

and the solution of Eq. 31 is:

$$\begin{bmatrix} P_{\mathcal{C}}(s) \\ P_{\mathcal{U}}(s) \end{bmatrix} = \frac{1}{\lambda+\mu} \begin{bmatrix} \lambda e^{-(\lambda+\mu)s} + \mu & \mu(1-e^{-(\lambda+\mu)s}) \\ \lambda(1-e^{-(\lambda+\mu)s}) & \lambda + \mu e^{-(\lambda+\mu)s} \end{bmatrix} \begin{bmatrix} P_{\mathcal{C}}(0) \\ P_{\mathcal{U}}(0) \end{bmatrix}. \quad (32)$$

The simulation procedure was as follows: we initialized $X_i(0)$ in one of the states C or U. We used Euler's scheme to discretize Eq. 28 at a time step Δt. For each consecutive step $k\Delta t$, $k = 1,...n_i$, we determined the state of the trajectory from the probabilities $P_C(\Delta t)$,$P_U(\Delta t)$ (Eq. 30), using the previous state as the initial condition:

$$\begin{bmatrix} P_{\mathcal{C}}(0) \\ P_{\mathcal{U}}(0) \end{bmatrix} = \begin{cases} [1,0]^T, & X_i((k-1)\Delta t) \in \mathcal{C}; \\ [0,1]^T, & X_i((k-1)\Delta t) \in \mathcal{U}. \end{cases} \quad (33)$$

3.2 Evaluating the classifier using ground-truth trajectories

To measure the accuracy of our classification algorithm (Sections 2.2-2.3), we generated N = 100 trajectories $Y_i(k\Delta t)$, i = 1,...,N, k = 1,...,n_i using Eq. 29, where the length of a trajectory n_i was chosen randomly from a Poisson distribution with average length n = 100 time points, Δt = 0.02s, κ = 2, different values of D_C and D_U (see below), and twenty equally spaced values of λ, $\mu \in [0,1]$ to obtain ground-truth states $S_i(k\Delta t) \in [C,U]$ associated with each trajectory $Y_i(k\Delta t)$. We used the four-parameter classifier (section 2.3) to classify the trajectories $Y_i(k\Delta t)$ and add a label $C_i(k\Delta t) \in [C, U]$. To evaluate the similarity between the true class set S_i and the output of the classification classes C_i, we used an indicator function $\delta_i(k\Delta t)$, defined by:

$$\delta_i(k) = \begin{cases} 1, & S_i(k\Delta t) = C_i(k\Delta t); \\ 0, & \text{else}, \end{cases} \quad (34)$$

and then used Eq. 34 to define a parameter M that measures the accuracy of the classification along all trajectories. It was defined by:

$$M = \frac{\sum_{i=1}^{N} \sum_{k=1}^{n_i} \delta_i(k)}{\sum_{i=1}^{N} n_i}. \quad (35)$$

We evaluated the accuracy M (Eq. 35) for an ensemble of trajectories generated with twenty values of $D_C \in [0.002, 0.1]$ and twenty values of $D_U \in [0.2, 1]$ $\mu m^2/s$, where we set the switching rates to be λ = 0.8, μ = 0.9): we found that the classifier achieved above 90% accuracy in the majority of the parameter

space, dropping to 76% for confined and unconfined diffusion constants where we restricted $D_C \in [0.02, 0.1]$, and $D_U \in [0.1, 0.2]$ [see **Supplementary Figure 1b** (left)]. In addition, to test the robustness of the classification algorithm to the switching rates, we evaluated M (Eq. 35) for twenty values of the switching rates $\lambda, \mu \in [0, 1]$ where D_C and D_U were similar (0.008 and 0.01 $\mu m^2/s$, respectively) and obtained a very good accuracy – above 85% for all values of λ, μ [see **Supplementary Figure 1b** (right)].

In summary, when tested on simulated data, the classification algorithm was able to robustly classify trajectories into confined and unconfined/sub-diffusive states.

4. Estimation of association and dissociation times from trajectories

After classifying the 20 ms data trajectories into two classes C and U (see section 2.3), we estimated the association and dissociation time constants as follows. The association time τ_A for trajectory X_i is defined as the time spent freely diffusing between confined chromatin-bound states:

$$t_A = \inf_t (t > 0; X_i(t) \in C \mid X_i(0) \in U). \quad (36)$$

The dissociation time τ_D is the time spent bound to chromatin between unconfined freely diffusing states:

$$t_D = \inf_t (t > 0; X_i(t) \in U \mid X_i(0) \in C). \quad (37)$$

To construct the histogram of these times, we reset the origin of time to zero after each association or dissociation event. In practice, for each partition of a trajectory X_i in a given ensemble, we collected the sequence τ_A^i of association times as the consecutive time points for which the trajectory spends in class U between being confined, as determined by the posterior probability P (Eq. 26). Equivalently, the dissociation times τ_D are those times in which the trajectory spends in confined state C between being unconfined. We fitted the histogram of association times and dissociation times with a single exponential[101]:

$$f_A(t) = \beta_D \exp(-\mu t), \text{ and} \quad (38)$$

$$f_D(t) = \beta_A \exp(-\lambda t). \qquad (39)$$

The association and dissociation times are then obtained by $\langle \tau_A \rangle = \frac{1}{\mu}$ and $\langle \tau_D \rangle = \frac{1}{\lambda}$.

5. Gaussian fitting of 500 ms exposure anomalous exponents

Although the algorithm we designed segments 500 ms exposure trajectories into slow-diffusing and fast-diffusing components, the distribution of the anomalous exponents in the fast-diffusing component showed that two peaks can clearly be identified by Gaussian fitting. Indeed, when we fitted 1, 2 or 3 Gaussians to the anomalous exponent distributions for chromatin bound NuRD complex subunits such as CHD4, MBD3 and MTA2 in wild-type ES cells, we found that 2 Gaussians were the best minimal model to account for the data – having the lowest BIC value (see Eq. 13 and **Extended Data Figure 5d**). This result was supported by the initial displacement analysis that we carried out (see Section 1), which also suggested that the fast components can be decomposed into two sub-populations.

To ensure enough data had been collected to account for cell-to-cell heterogeneity, data was collated from 3 fields of view with around 6 cells in each field of view imaged, leading to a total of around 18 cells per condition. To assess the reproducibility of these results an additional 3 fields of view containing around 18 cells were collected for chromatin bound CHD4 on a different day and shown to have a similar anomalous exponent distribution (data not shown). Single molecule imaging of CHD4 in the *Mbd3*-ko was also shown to have a significantly different distribution when compared to both wild-type ES cell CHD4 datasets (p = 0.005 and p = 0.02 respectively).

6. Instructions for use of the code for classification and segmentation of trajectories into two diffusion states using four parameters (4P)

The code used for classification and segmentation of trajectories into two diffusion states using four parameters (4P) is available at: https://zenodo.org/record/6497411#.YmlGFy8w3q0. Click "Run" on MATLAB and then select all the relevant input files to include in the classification. The input format is a .csv file with the following columns:

Track number	frame	x	y	z

The input parameters to define are as follows:

1. Exposure time for a frame e.g. 0.02 for 20 ms or 0.5 for 500 ms
    - `'dt',0.5,...                        % aquisition time interval [sec]`
2. Options to exclude short trajectories. This parameter also specifies the minimal length that a molecule must spend confined/unconfined for that sub trajectory to contribute to association/dissociation time calculations:
    - `'minNumPoints',2,...               % (frames) threshold below which trajectories are discarded`
3. Option to deal with misclassification errors for accurate final parameter calculation. To increase the accuracy of the calculation, we say that the period a molecule continuously spends confined/unconfined must be at least 7 frames long, i.e. at least 7 adjacent sliding windows must be assigned to the same class:
    - `'minNumPointsLongTraj',7,...    % minimal number of frames to be considered as non-confined long trajectory`
4. Number of frames to include in the sliding window either side of a point on the trajectory
    - `'numMSDpoints',5,...             % radius (number of points) to consider in the moving window around each point`
    - For this study, we used optimised parameters of 5 frames before and after (11 frames in total)
    - Trade-off: longer sliding windows give better estimates of the parameters, but fail to detect faster transitions
5. Option to classify by all 4 parameters or to choose a subset of them.
    - `'classifyUsingAlpha', true,...     %use anomalous exponent in classification`

- `'classifyUsingDriftNorm',true,... % use drift norm in classification`
- `'classifyUsingDiffusion',true,... % use diffusion constant in classification`
- `'classifyUsingLc', true,...    % use Lc in classification`
- `'classifyUsingSpringConst', false,... % use Kc in classification`

We currently do not classify by Kc, a spring constant useful for some analyses, but this is included in the code in case it is of use to others.

To generate outputs, set the following export function to true:

- `'exportResults', true,...      % export results  [true/false]`

This will output the following:

1. .csv files for biophysical parameters calculated for sliding windows in each trajectory (see Supplementary Table 6):

2. .csv files for each biophysical parameter calculated for periods spent confined (note that this is the average parameter for a period spent confined, so if molecules spend a longer time confined, the estimate is based on values from more sliding windows)
3. .csv files for each biophysical parameter calculated for periods spent unconfined
4. .csv file of association times (time spent unconfined between periods spent confined) – this excludes times where a molecule transitions for <10 frames as this may be a misclassification
5. .csv file for dissociation times (time spent confined between periods spent unconfined) – this excludes times where a molecule transitions for <10 frames as this may be a misclassification

Additional outputs (set to 'true' by default) include:

6. Visualisation function in MATLAB to see all 3D trajectories but also all confined/unconfined trajectories outputted (sub-trajectory segments are coloured blue vs orange for confined vs unconfined segments).
   a. `'plotTrajectories',true,...    % show all 3d trajectories [true/false]`
   b. `'inspectTrajectories',true,... % allows to inspect visually all, confined and unconfined trajectories`

7. Options to plot the histograms of statistical parameters, association/dissociation times and trajectory duration times:

   a. `'plotStatisticalParameters',true,... % show the distribution of statistical parameters`
   b. `'plotTrajDurationHist',true,...% plot trajectory duration histogram [true/false]`
   c. `'plotAssociationDissociationHist',true,...% show the association dissociation histogram`

8. Option to save figures of histograms outputted in Option 7

   a. `'exportFigures',true,...       % export fig files`

9. Export trajectories as PDB files to generate figures

   a. `'exportPDBtrajectories', true,... % export trajectories as pdb file`

10. Changing the number of histogram fitting for association/dissociation times (default is 5)

    a. `'numHistFitTrials',5,...         % number histogram fitting trials`

11. Option to classify by more than 2 Gaussian distributions (again not used in the paper but left as an option for other groups)

    a. `'numberOfClasses',2,...              % number of classes (confined/unconfined/others..)`
