# Supplementary material for: Live-cell three-dimensional single-molecule tracking reveals modulation of enhancer dynamics by NuRD
Source: Nat Struct Mol Biol. 2023 Sep 28;30(11):1628–39. doi: 10.1038/s41594-023-01095-4 (PMC10643137; doi:10.1038/s41594-023-01095-4)
Supplement: Supplementary file 2 — Reporting Summary [file 41594_2023_1095_MOESM2_ESM.pdf]

nature research

Corresponding author(s): B D Hendrich, D Klenerman, D Holcman and E D Laue

Last updated by author(s): Jun 27, 2023

Reporting Summary

Nature Research wishes to improve the reproducibility of the work that we publish. This form provides structure for consistency and transparency in reporting. For further information on Nature Research policies, see Authors & Referees and the Editorial Policy Checklist.

Statistics

For all statistical analyses, confirm that the following items are present in the figure legend, table legend, main text, or Methods section.

n/a	Confirmed	
☐	☒	The exact sample size (*n*) for each experimental group/condition, given as a discrete number and unit of measurement
☐	☒	A statement on whether measurements were taken from distinct samples or whether the same sample was measured repeatedly
☐	☒	The statistical test(s) used AND whether they are one- or two-sided<br>*Only common tests should be described solely by name; describe more complex techniques in the Methods section.*
☒	☐	A description of all covariates tested
☐	☒	A description of any assumptions or corrections, such as tests of normality and adjustment for multiple comparisons
☐	☒	A full description of the statistical parameters including central tendency (e.g. means) or other basic estimates (e.g. regression coefficient) AND variation (e.g. standard deviation) or associated estimates of uncertainty (e.g. confidence intervals)
☐	☒	For null hypothesis testing, the test statistic (e.g. *F*, *t*, *r*) with confidence intervals, effect sizes, degrees of freedom and *P* value noted<br>*Give P values as exact values whenever suitable.*
☒	☐	For Bayesian analysis, information on the choice of priors and Markov chain Monte Carlo settings
☒	☐	For hierarchical and complex designs, identification of the appropriate level for tests and full reporting of outcomes
☒	☐	Estimates of effect sizes (e.g. Cohen's *d*, Pearson's *r*), indicating how they were calculated

Our web collection on statistics for biologists contains articles on many of the points above.

Software and code

Policy information about availability of computer code

Data collection

Imaging data collection: Micro-manager (https://www.micro-manager.org) and ImageJ (https://imagej.nih.gov/ij/)

Data analysis

See Methods for references:

Hi-C analysis: NucProcess (https://github.com/tjs23/nuc_processing); NucTools (https://github.com/tjs23/nuc_tools); Juicer (https://github.com/aidenlab/juicer); Cooler (https://cooler.readthedocs.io/en/latest/); CscoreTool (https://github.com/scoutzxb/CscoreTool); lavaburst (https://github.com/nvictus/lavaburst); enhancer-promoter analysis (https://github.com/dhall1995/Acitivity-By-Contact_Enhancer-Promoter_Link_Prediction);

Cut&Run analysis: Trim galore (https://www.bioinformatics.babraham.ac.uk/projects/trim_galore/); Bowtie2 (REF. 74); Deeptools v2.5.0 (https://github.com/deeptools/deepTools).

Single molecule/foci image analysis: [PeakFit (REF. 99), easy-DHPSF software (REF. 79)]; Trajectory analysis (https://github.com/wb104/trajectory-analysis); Gaussian mixture model classification (https://zenodo.org/record/6497411#.YmlGFy8w3q0);

3D DNA FISH analysis: Imaris 9.6.

For manuscripts utilizing custom algorithms or software that are central to the research but not yet described in published literature, software must be made available to editors/reviewers. We strongly encourage code deposition in a community repository (e.g. GitHub). See the Nature Research guidelines for submitting code & software for further information.

Data

Policy information about availability of data

All manuscripts must include a data availability statement. This statement should provide the following information, where applicable:

- Accession codes, unique identifiers, or web links for publicly available datasets
- A list of figures that have associated raw data
- A description of any restrictions on data availability

The single-molecule/locus imaging movies and XYZt single molecule/locus trajectory data files are available at: https://zenodo.org/deposit/7985268. (DOI: 10.5281/zenodo.7985268)

Figures 1, 2, 3 and 4, and Extended Data Figures 2, 3, 4, 5 and 7 - XYZt data

Cut&Run and Hi-C datasets are available from the Gene Expression Omnibus (GEO) repository under accession code GSE179007. These data were processed using the GRCm38.p6 mouse reference genome (https://www.ncbi.nlm.nih.gov/assembly/GCF_000001635.26/).

Extended Data Figure 9 - Cut&Run data

Figure 5 and Extended Data Figures 8 and 10 - Hi-C data

Field-specific reporting

Please select the one below that is the best fit for your research. If you are not sure, read the appropriate sections before making your selection.

☒ Life sciences ☐ Behavioural & social sciences ☐ Ecological, evolutionary & environmental sciences

For a reference copy of the document with all sections, see nature.com/documents/nr-reporting-summary-flat.pdf

Life sciences study design

All studies must disclose on these points even when the disclosure is negative.

Sample size

In Figure 2c and Extended Data Figure 3c, the apparent diffusion coefficient of freely diffusing CHD4 and MTA2 molecules increase upon removal of MBD3. We ensured a minimum of 80 trajectories to estimate the diffusion coefficient for MTA2 molecules since our data indicate most MTA2 molecules are associated with the remodeller (shift in diffusion coefficient histogram observed as opposed to change in variance upon removal of MBD3). In contrast, because CHD4 exists on its own as well as in the NuRD (~36 %) and ChAHP (~7 %) complexes, we recorded > 900 trajectories per CHD4 sample to ensure detection of changes in diffusion coefficient for CHD4 in complexes that may be as little as ~36 % of the total CHD4 molecules. In Figure 2c and Extended Data Figure 3c, the percentage of freely diffusing CHD4 and MTA2 molecules (as opposed to chromatin bound) increased in the absence of MBD3 by 5 % and 36 % respectively. We ensured these percentages were represented by at least 100 trajectories. For example, to determine the small 5 % increase for CHD4, > 2000 trajectories per sample is sufficient (5 % is >100 trajectories). To determine the 36 % increase of MTA2, >300 trajectories is sufficient (36 % is >100 trajectories). In Figure 2d, our data indicated a single exponential fit and so we ensured > 60 association times were sufficient to accurately fit association times. In Figure 3 and Extended Data Figure 5, we analyse changes in the movement of chromatin bound CHD4. Since the F2 state accounted for as low as 7 % of the total trajectories and since as little as 36 % of CHD4 could be in NuRD as opposed to other complexes, we ensured >4500 trajectories to ensure more than 100 trajectories of the bound NuRD complex were used to estimate parameters in the F2 state.

Data exclusions

No data were excluded.

Replication

Replicates were collected for all experiments, and all were successful. Biochemical experiments are representative of ≥3 independent replicates. Overlap of Hi-C replicates and comparison with published replicates is shown in Extended Data Figure 8a. Overlap of CTCF/SMC3 Cut&Run replicates is shown in Extended Data Figures 9a and 9b. Replicates were collected for all single-molecule tracking datasets and are as described in "Live-cell 3D single-molecule imaging" in the Methods, or in "Gaussian fitting of 500 ms exposure anomalous exponents" in the Supplementary Methods. "Data was collated from 3 fields of view with either around 3 or 6 cells in each field of view imaged, leading to a total of around 9 or 18 cells per condition." In addition, "to assess the reproducibility of these results an additional 3 fields of view containing around 18 cells were collected for chromatin bound CHD4 on a different day and shown to have a similar anomalous exponent distribution".

Randomization

N/A - sample allocation was not randomized because comparative experiments were designed, carried out and analysed by the same person.

Blinding

N/A - see above.

Reporting for specific materials, systems and methods

We require information from authors about some types of materials, experimental systems and methods used in many studies. Here, indicate whether each material, system or method listed is relevant to your study. If you are not sure if a list item applies to your research, read the appropriate section before selecting a response.

Materials & experimental systems

n/a	Involved in the study
☐	☒ Antibodies
☐	☒ Eukaryotic cell lines
☒	☐ Palaeontology
☒	☐ Animals and other organisms
☒	☐ Human research participants
☒	☐ Clinical data

Methods

n/a	Involved in the study
☐	☒ ChIP-seq
☒	☐ Flow cytometry
☒	☐ MRI-based neuroimaging

Antibodies

Antibodies used

Antibodies used were:

1. Rabbit anti-CTCF, Millipore, 07-729, Polyclonal
2. Rabbit anti-SMC3, Abcam, ab9263, Polyclonal
3. Mouse anti-CHD4, Abcam, ab70469, Monoclonal [3F2/4]
4. Mouse anti-FLAG, Sigma, F1804, Monoclonal [M2]
5. Rabbit anti-GATAD2A, Abcam, ab87663, Polyclonal
6. Rabbit anti-HDAC1, Abcam, ab7028, Polyclonal
7. Rabbit anti-MBD3, Abcam, ab157464, Monoclonal [EPR9913]
8. Mouse anti-MTA2, Abcam, ab50209, Monoclonal [MTA2-276]
9. Mouse anti-PCNA, Santa Cruz, Sc56, Monoclonal [PC10]
10. Recombinant GFP-Booster Alexa Fluor® 488 nanobody, ChromoTek, gb2AF488

Validation

Primary antibodies validated in multiple previous studies. Knockout validation examples are provided below:

1. Rabbit anti-CTCF - (Ren et al, Mol Cell. 2017 Sep 21;67(6):1049-1058.e6. doi: 10.1016/j.molcel.2017.08.026)
2. Rabbit anti-SMC3 - (Wang et al, Exp Hematol 2019 70:70-84.e6)
3. Mouse anti-CHD4 - (O'Shaughnessy-Kirwan et al, Development. 2015 Aug 1; 142(15): 2586–2597)
4. Mouse anti-FLAG - validated in manuscript as negative using untagged cell lines
5. Rabbit anti-GATAD2A - validated using knock-down cells and purified GATAD2A.
6. Rabbit anti-HDAC1 - (Gonneaud et al, Sci Rep. 2019 Mar 29;9(1):5363. doi: 10.1038/s41598-019-41842-6)
7. Rabbit anti-MBD3 - validated in manuscript as negative in Mbd3-knockout cell lines and in (Bornelov et al, Mol Cell. 2018 Jul 5; 71(1): 56–72.e4)
8. Mouse anti-MTA2 - (Burgold et al, EMBO J. 2019 Jun 17; 38(12): e100788)
9. Mouse anti-PCNA - (Dietsch et al, Biotechniques 2017 Feb 1;62(2):80-82. doi: 10.2144/000114518)

Eukaryotic cell lines

Policy information about cell lines

Cell line source(s)

Sf21 insect cells were used for the expression of recombinant proteins.

The background mouse E14tg2a ES cells are available from Sigma Aldrich (08021401). Cell lines generated in this study are described in "Mouse embryonic stem cell line generation" and are being deposited at Addgene.

Authentication

The background mouse E14tg2a ES cell lines and those generated in this study were characterized by qPCR, RNA-seq, ChIP-seq, and potency assays. In addition, Western blots and immunoprecipitation studies of the NuRD complex are shown in Extended Data Figure 1.

Mycoplasma contamination

Mouse ES cell lines used in this study were routinely screened for mycoplasma contamination and tested negative.

Commonly misidentified lines
(See ICLAC register)

None were used in this study.

ChIP-seq

Data deposition

☒ Confirm that both raw and final processed data have been deposited in a public database such as GEO.

☒ Confirm that you have deposited or provided access to graph files (e.g. BED files) for the called peaks.

Data access links
May remain private before publication.

https://www.ncbi.nlm.nih.gov/geo/query/acc.cgi?acc=GSE179007

Files in database submission

Processed data files:
SLX-20518.A1.HCCJGDRXY.Q30.srt.nodup.noChrM.bam_peaks.narrowPeak.txt.gz

SLX-20518.A4.HCCJGDRXY.Q30.srt.nodup.noChrM.bam_peaks.narrowPeak.txt.gz
SLX-20518.A6.HCCJGDRXY.Q30.srt.nodup.noChrM.bam_peaks.narrowPeak.txt.gz
SLX-20518.B2.HCCJGDRXY.Q30.srt.nodup.noChrM.bam_peaks.narrowPeak.txt.gz
SLX-20518.B4.HCCJGDRXY.Q30.srt.nodup.noChrM.bam_peaks.narrowPeak.txt.gz
SLX-20518.B7.HCCJGDRXY.Q30.srt.nodup.noChrM.bam_peaks.narrowPeak.txt.gz
SLX-20518.E1.HCCJGDRXY.Q30.srt.nodup.noChrM.bam_peaks.narrowPeak.txt.gz
SLX-20518.E3.HCCJGDRXY.Q30.srt.nodup.noChrM.bam_peaks.narrowPeak.txt.gz
SLX-20518.E6.HCCJGDRXY.Q30.srt.nodup.noChrM.bam_peaks.narrowPeak.txt.gz
SLX-20518.F1.HCCJGDRXY.Q30.srt.nodup.noChrM.bam_peaks.narrowPeak.txt.gz
SLX-20518.F4.HCCJGDRXY.Q30.srt.nodup.noChrM.bam_peaks.narrowPeak.txt.gz
SLX-20518.F6.HCCJGDRXY.Q30.srt.nodup.noChrM.bam_peaks.narrowPeak.txt.gz
SLX-20518.A1.HCCJGDRXY.Q30.srt.nodup.noChrM.bam_treat_pileup_filter_norm.bw.txt.gz
SLX-20518.A4.HCCJGDRXY.Q30.srt.nodup.noChrM.bam_treat_pileup_filter_norm.bw.txt.gz
SLX-20518.A6.HCCJGDRXY.Q30.srt.nodup.noChrM.bam_treat_pileup_filter_norm.bw.txt.gz
SLX-20518.B2.HCCJGDRXY.Q30.srt.nodup.noChrM.bam_treat_pileup_filter_norm.bw.txt.gz
SLX-20518.B4.HCCJGDRXY.Q30.srt.nodup.noChrM.bam_treat_pileup_filter_norm.bw.txt.gz
SLX-20518.B7.HCCJGDRXY.Q30.srt.nodup.noChrM.bam_treat_pileup_filter_norm.bw.txt.gz
SLX-20518.E1.HCCJGDRXY.Q30.srt.nodup.noChrM.bam_treat_pileup_filter_norm.bw.txt.gz
SLX-20518.E3.HCCJGDRXY.Q30.srt.nodup.noChrM.bam_treat_pileup_filter_norm.bw.txt.gz
SLX-20518.E6.HCCJGDRXY.Q30.srt.nodup.noChrM.bam_treat_pileup_filter_norm.bw.txt.gz
SLX-20518.F1.HCCJGDRXY.Q30.srt.nodup.noChrM.bam_treat_pileup_filter_norm.bw.txt.gz
SLX-20518.F4.HCCJGDRXY.Q30.srt.nodup.noChrM.bam_treat_pileup_filter_norm.bw.txt.gz
SLX-20518.F6.HCCJGDRXY.Q30.srt.nodup.noChrM.bam_treat_pileup_filter_norm.bw.txt.gz

Raw data files:
SLX-20518.A1.HCCJGDRXY.s_2.r_1.fq.gz
SLX-20518.A1.HCCJGDRXY.s_2.r_2.fq.gz
SLX-20518.A4.HCCJGDRXY.s_2.r_1.fq.gz
SLX-20518.A4.HCCJGDRXY.s_2.r_2.fq.gz
SLX-20518.A6.HCCJGDRXY.s_2.r_1.fq.gz
SLX-20518.A6.HCCJGDRXY.s_2.r_2.fq.gz
SLX-20518.B2.HCCJGDRXY.s_2.r_1.fq.gz
SLX-20518.B2.HCCJGDRXY.s_2.r_2.fq.gz
SLX-20518.B4.HCCJGDRXY.s_2.r_1.fq.gz
SLX-20518.B4.HCCJGDRXY.s_2.r_2.fq.gz
SLX-20518.B7.HCCJGDRXY.s_2.r_1.fq.gz
SLX-20518.B7.HCCJGDRXY.s_2.r_2.fq.gz
SLX-20518.D3.HCCJGDRXY.s_2.r_1.fq.gz
SLX-20518.D3.HCCJGDRXY.s_2.r_2.fq.gz
SLX-20518.D5.HCCJGDRXY.s_2.r_1.fq.gz
SLX-20518.D5.HCCJGDRXY.s_2.r_2.fq.gz
SLX-20518.D8.HCCJGDRXY.s_2.r_1.fq.gz
SLX-20518.D8.HCCJGDRXY.s_2.r_2.fq.gz
SLX-20518.E1.HCCJGDRXY.s_2.r_1.fq.gz
SLX-20518.E1.HCCJGDRXY.s_2.r_2.fq.gz
SLX-20518.E3.HCCJGDRXY.s_2.r_1.fq.gz
SLX-20518.E3.HCCJGDRXY.s_2.r_2.fq.gz
SLX-20518.E6.HCCJGDRXY.s_2.r_1.fq.gz
SLX-20518.E6.HCCJGDRXY.s_2.r_2.fq.gz
SLX-20518.F1.HCCJGDRXY.s_2.r_1.fq.gz
SLX-20518.F1.HCCJGDRXY.s_2.r_2.fq.gz
SLX-20518.F4.HCCJGDRXY.s_2.r_1.fq.gz
SLX-20518.F4.HCCJGDRXY.s_2.r_2.fq.gz
SLX-20518.F6.HCCJGDRXY.s_2.r_1.fq.gz
SLX-20518.F6.HCCJGDRXY.s_2.r_2.fq.gz
SLX-20518.H2.HCCJGDRXY.s_2.r_1.fq.gz
SLX-20518.H2.HCCJGDRXY.s_2.r_2.fq.gz
SLX-20518.H5.HCCJGDRXY.s_2.r_1.fq.gz
SLX-20518.H5.HCCJGDRXY.s_2.r_2.fq.gz
SLX-20518.H7.HCCJGDRXY.s_2.r_1.fq.gz
SLX-20518.H7.HCCJGDRXY.s_2.r_2.fq.gz

Genome browser session
(e.g. UCSC)

No longer applicable.

Methodology

Replicates

Three biological replicates were obtained per Cut&Run sample. They showed good agreement in peaks called as indicated in Extended Data Figures 9a and 9b.

Sequencing depth

See "Cut&Run" in the Methods and Extended Data Figure 9a. 50 bp paired-end sequencing was carried out:

Samples: Total number of uniquely mapped reads (8-16 million reads/replicate)
Inputs: Total number of uniquely mapped reads (8-23 million reads/replicate)

Antibodies

Antibodies were:
1. Rabbit anti-CTCF, Millipore, 07-729, Polyclonal
2. Rabbit anti-SMC3, Abcam, ab9263, Polyclonal

Peak calling parameters

Read mapping:
All Cut&Run data was trimmed using trim_galore (https://www.bioinformatics.babraham.ac.uk/projects/trim_galore/) and then aligned (with standard parameters) using Bowtie2 (REF. 74) to the Mus Musculus reference genome GRCm38.p6 (https://www.ncbi.nlm.nih.gov/assembly/GCF_000001635.26/)

Peak calling:
Peaks were called using MACS2 (REF. 76) to be at FDR 1 % and above 5-fold enrichment.

Data quality

Peaks at FDR 1% and above 5-fold enrichment:

CTCF 0h 1 = 53716 peaks
CTCF 0h 2 = 61352 peaks
CTCF 0h 3 = 67375 peaks
Shared between replicates: 37868 peaks

CTCF 48h 1 = 66506 peaks
CTCF 48h 2 = 79875 peaks
CTCF 48h 3 = 66309 peaks
Shared between replicates: 47042 peaks

Smc3 0h 1 = 50641 peaks
Smc3 0h 2 = 49843 peaks
Smc3 0h 3 = 19356 peaks
Shared between replicates: 15167 peaks

Smc3 48h 1 = 37661 peaks
Smc3 48h 2 = 42628 peaks
Smc3 48h 3 = 53379 peaks
Shared between replicates: 25324 peaks

Data quality ensured by comparison of peaks called between replicates but also by comparing peaks to published datasets.

Software

See "Cut&Run" in the Methods section. Software used:

1) Trim galore (https://www.bioinformatics.babraham.ac.uk/projects/trim_galore/)
2) Bowtie2 (REF. 74)
3) Deeptools v2.5.0 (REF. 75)
4) MACS2 (REF. 76)
