# Supplementary material for: Live-cell three-dimensional single-molecule tracking reveals modulation of enhancer dynamics by NuRD
Source: Nat Struct Mol Biol. 2023 Sep 28;30(11):1628–39. doi: 10.1038/s41594-023-01095-4 (PMC10643137; doi:10.1038/s41594-023-01095-4)

Extended data figure 1a

Western blots showing levels of NuRD components, probed with antibodies as indicated. Arrow shows relevant lane for figure

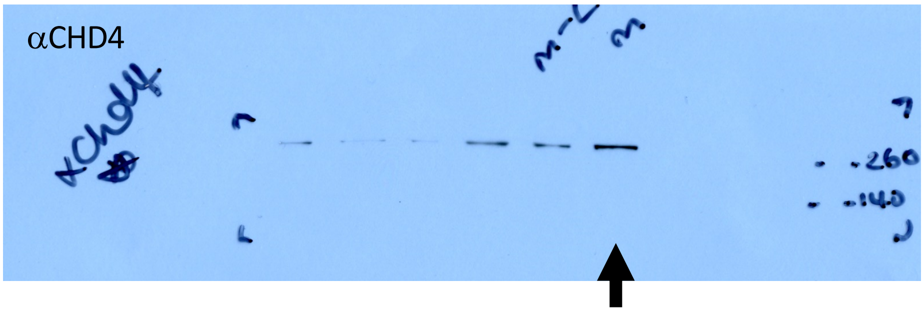

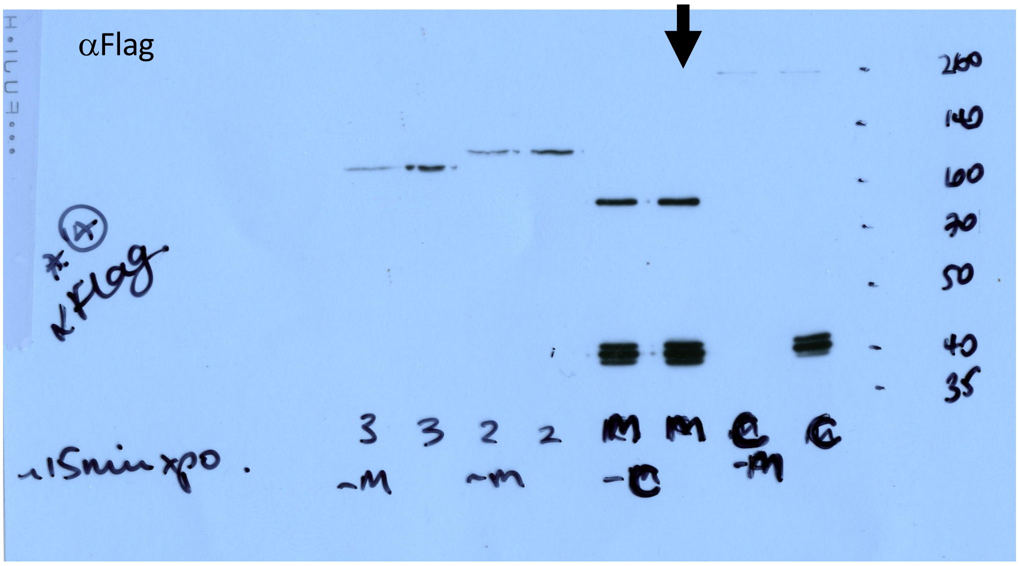

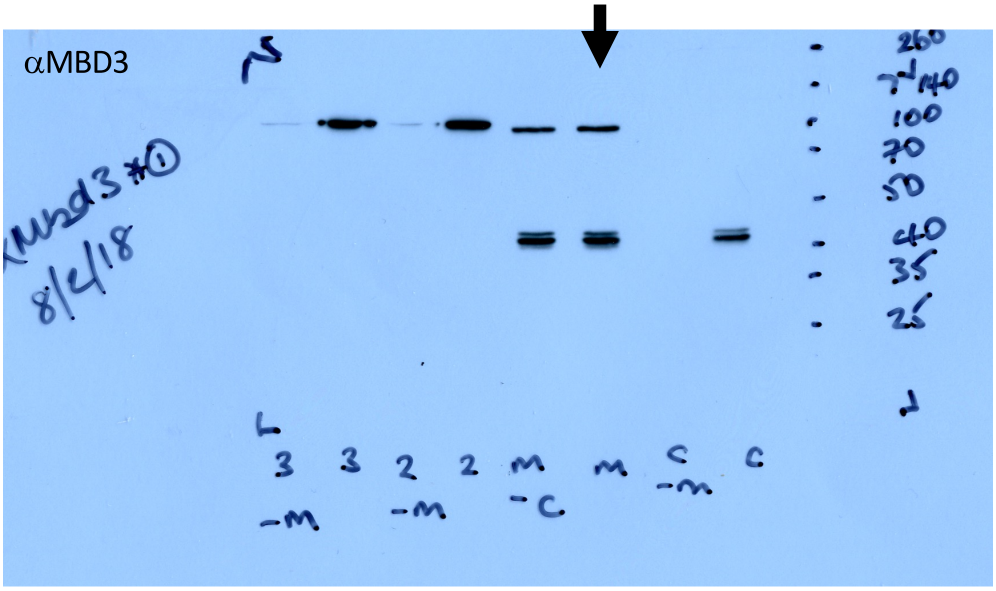

Extended data figure 1a continued

Western blots showing levels of NuRD components, probed with antibodies as indicated. Arrow shows relevant lane for figure

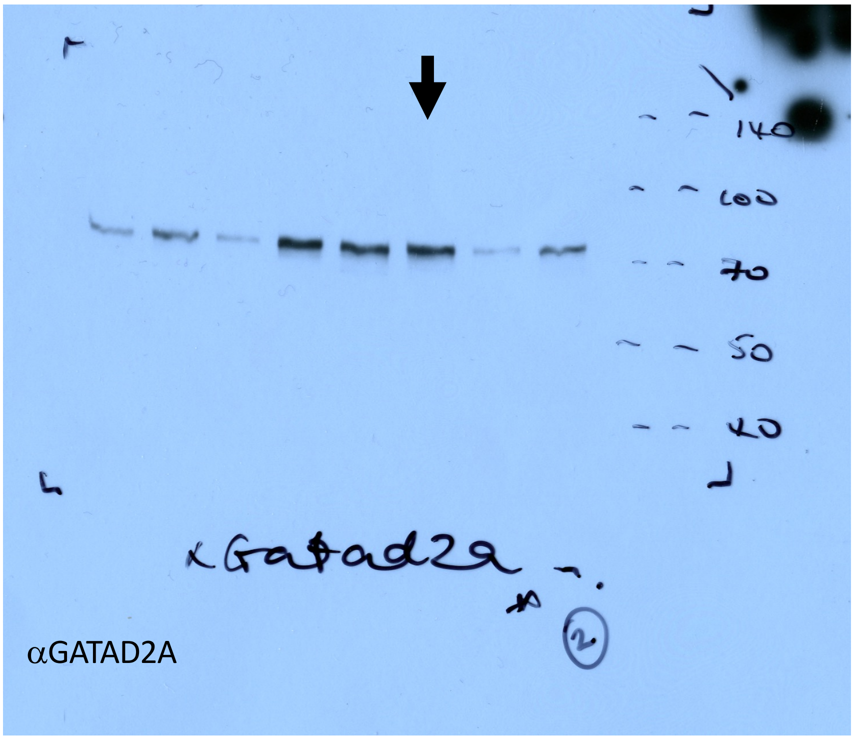

αGATAD2A

αHDAC1

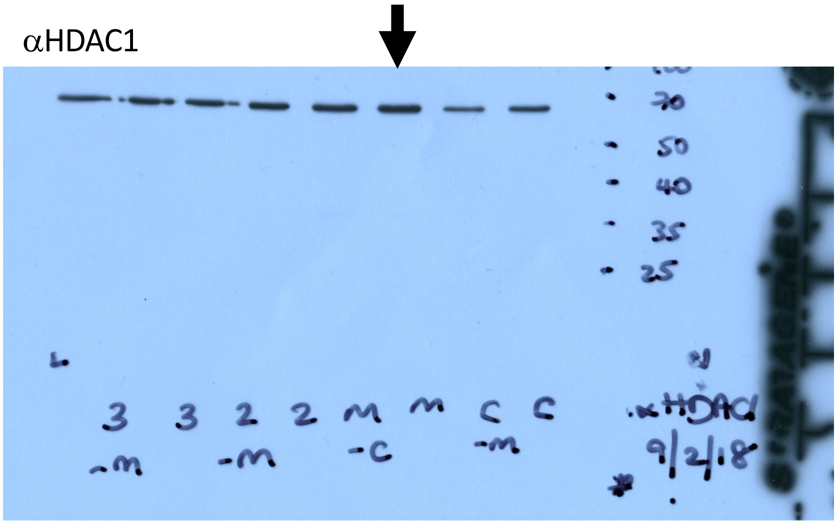

Basu et al

Extended data figure 1b

IP/Western blots probed with antibodies as indicated. Red bar shows relevant lanes.

αCHD4 IP/ Probed αCHD4

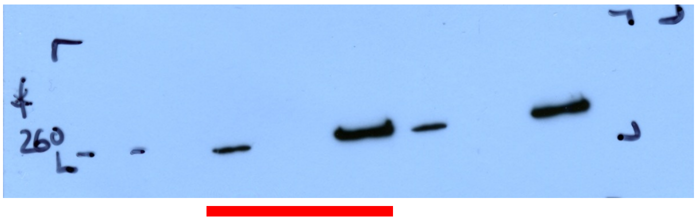

αCHD4 IP/ Probed αMTA2

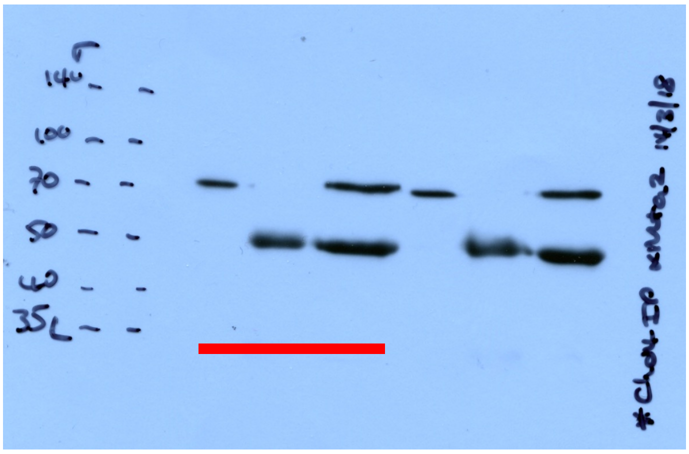

αCHD4 IP/ Probed αGATAD2A

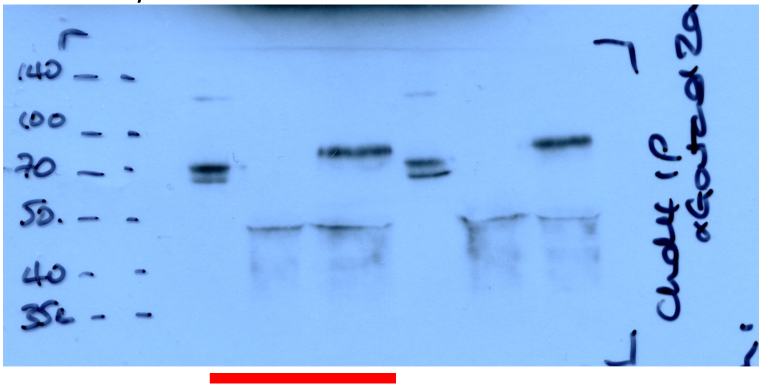

αCHD4 IP/ Probed αFlag

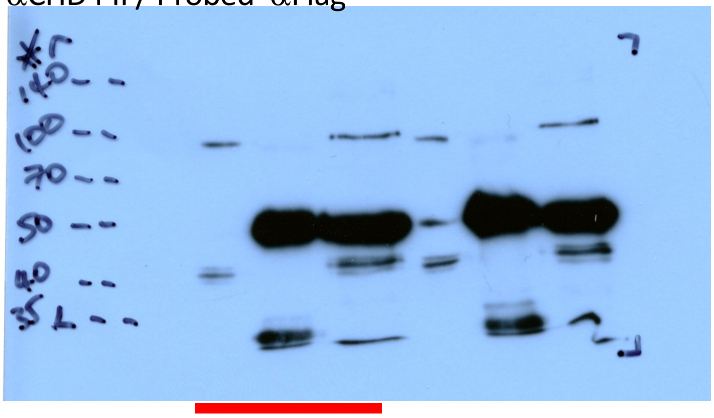

Basu et al

Extended data figure 1b continued

Western blots showing levels of NuRD components, probed with antibodies as indicated. Red box shows relevant lanes for figure

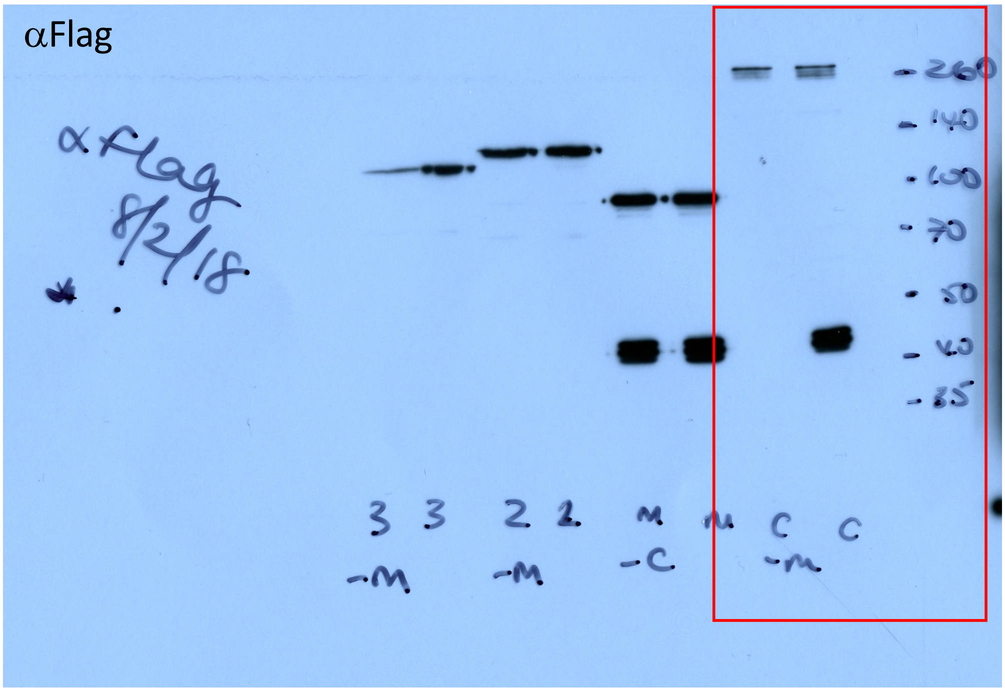

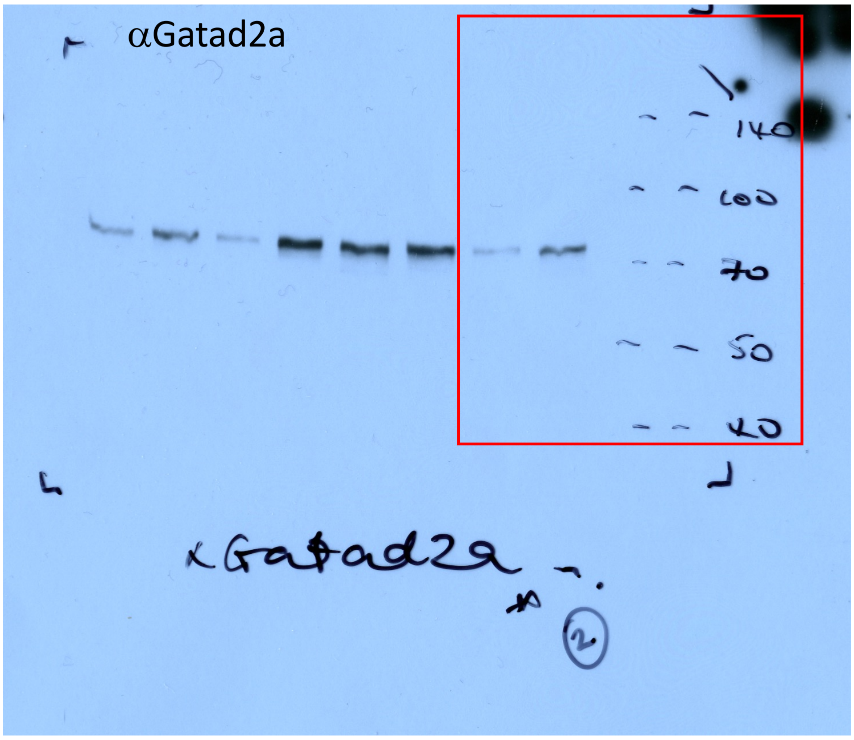

Basu et al

Extended data figure 1c

Western blots showing levels of NuRD components, probed with antibodies as indicated. Red box shows relevant lanes for figure

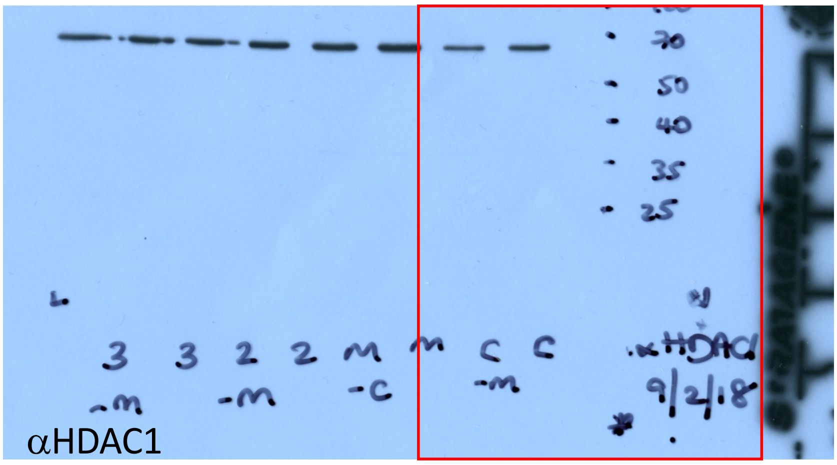

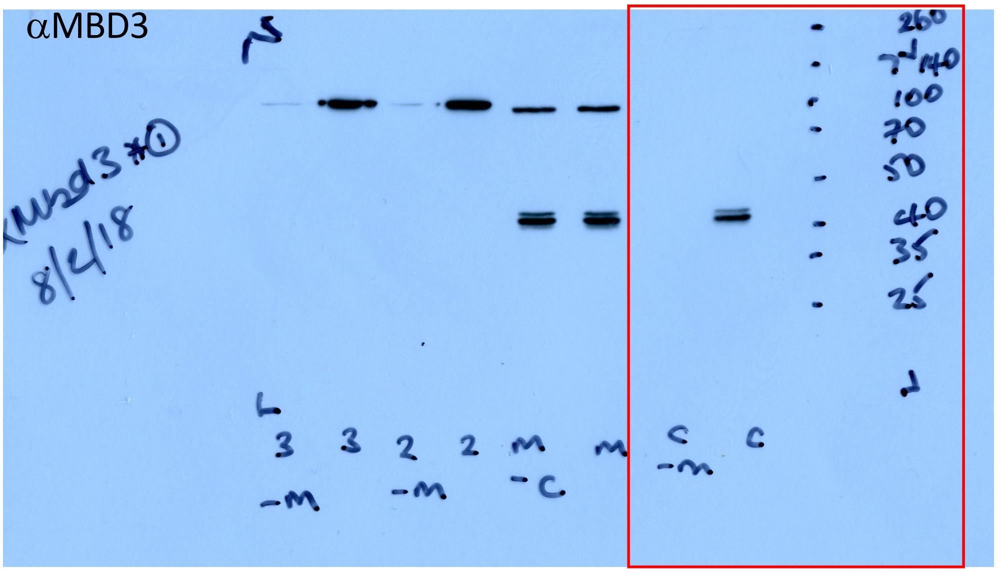

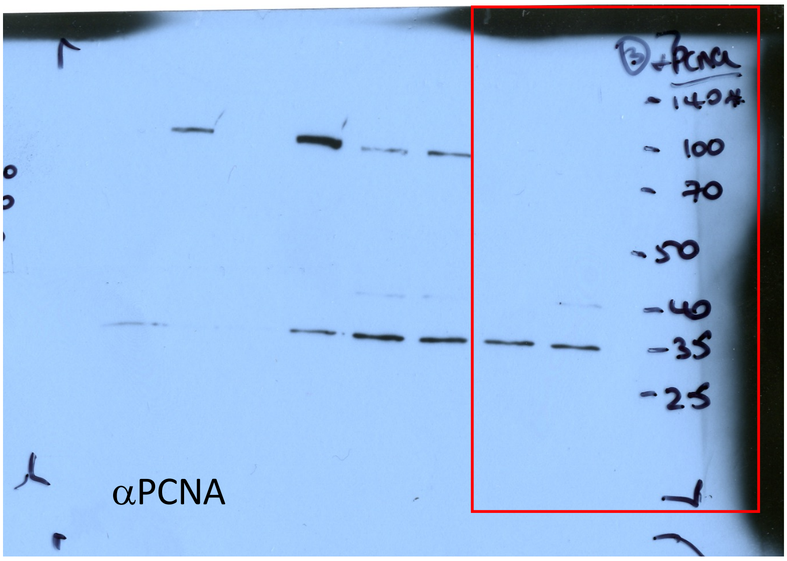

Extended data figure 1c continued

IP/Western blots probed with antibodies as indicated.

CHD4 Pull down / Probed αFlag

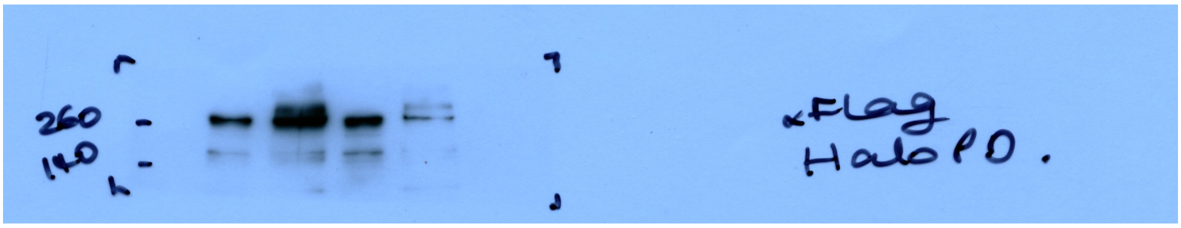

CHD4 Pull down / Probed αGATAD2A, αMBD3

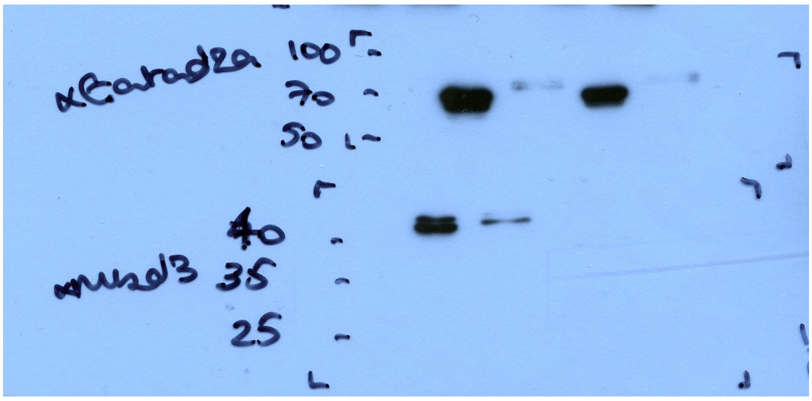

CHD4 Pull down / Probed αMTA2

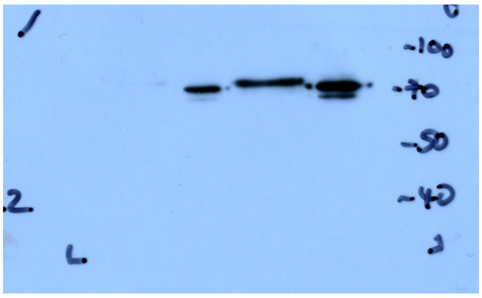

CHD4 Pull down / Probed αFlag/Gatad2a/Mbd3 (Duplicate experiment, two exposures)

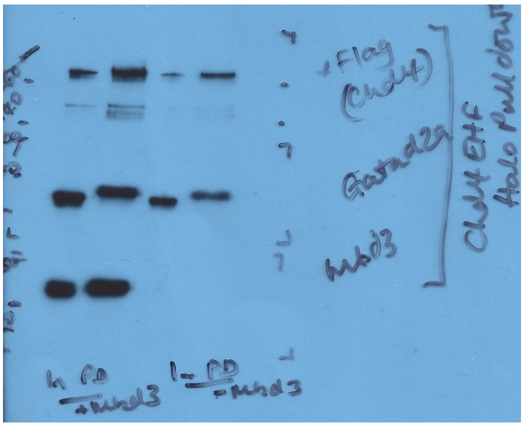

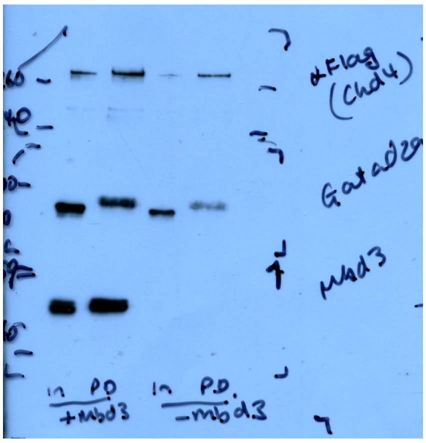

Basu et al

Extended data figure 1d

Western blots showing levels of NuRD components, probed with antibodies as indicated. Red box shows relevant lanes for figure

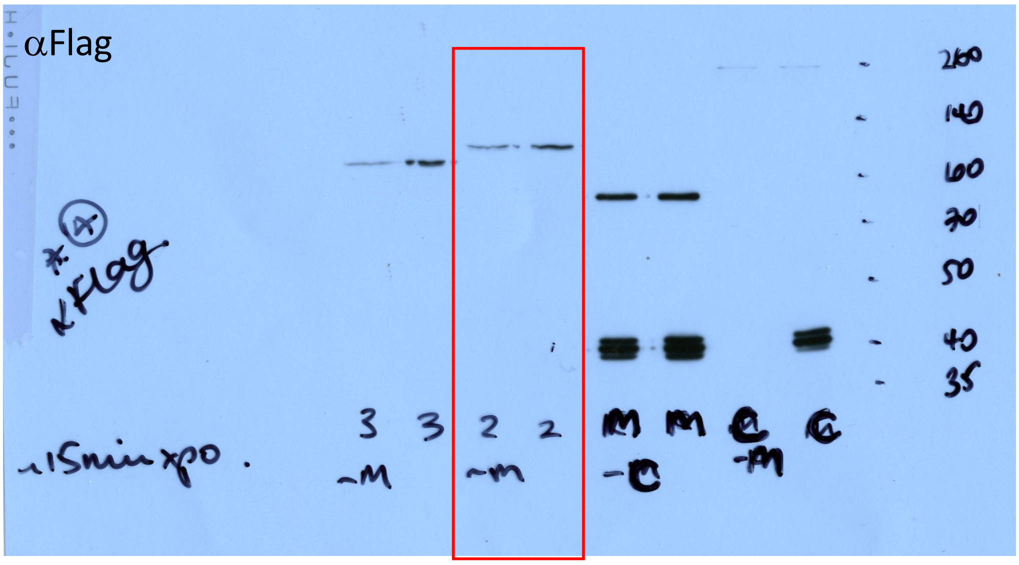

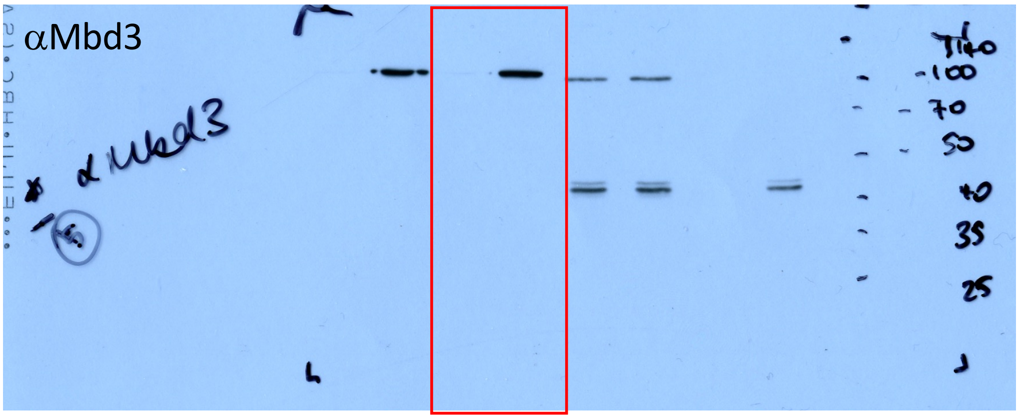

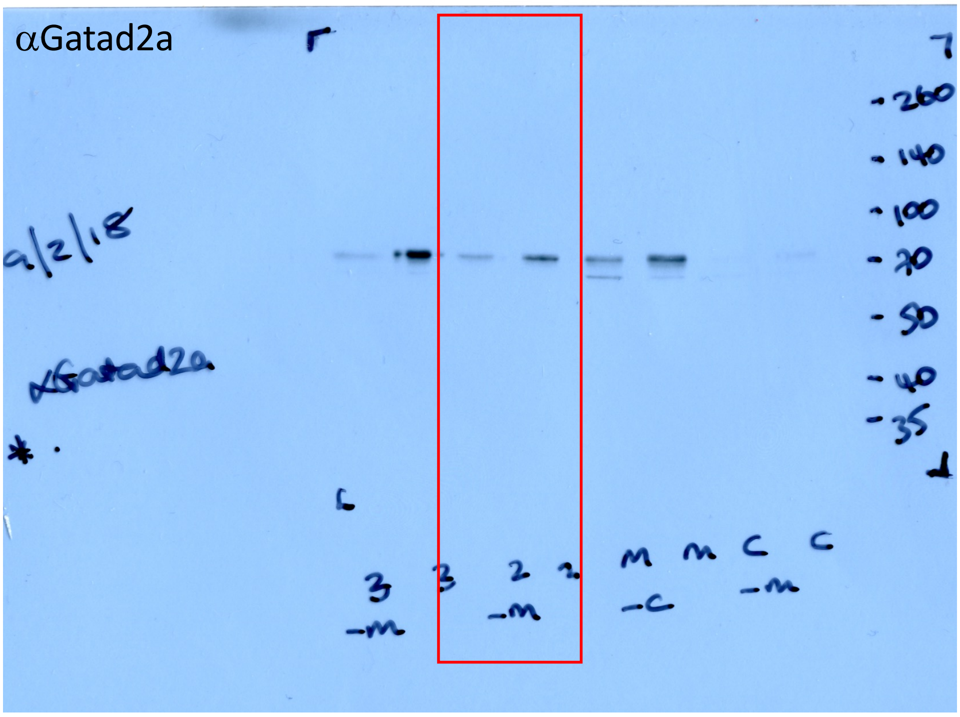

Extended data figure 1d continued

Western blots showing levels of NuRD components, probed with antibodies as indicated. Red box shows relevant lanes for figure

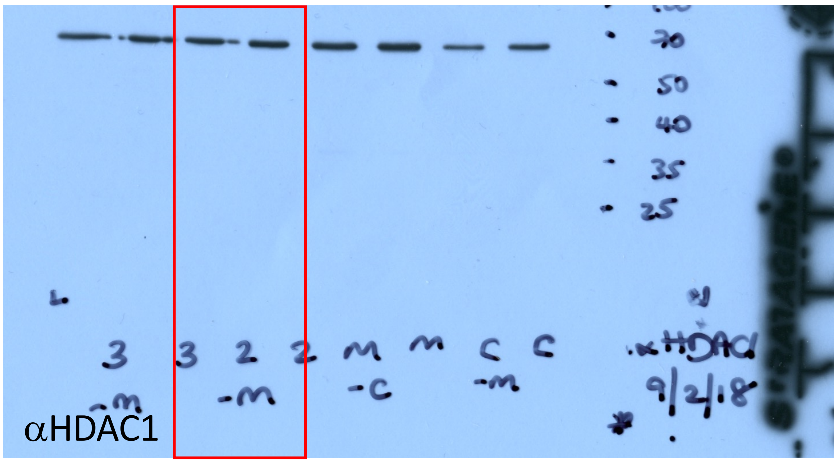

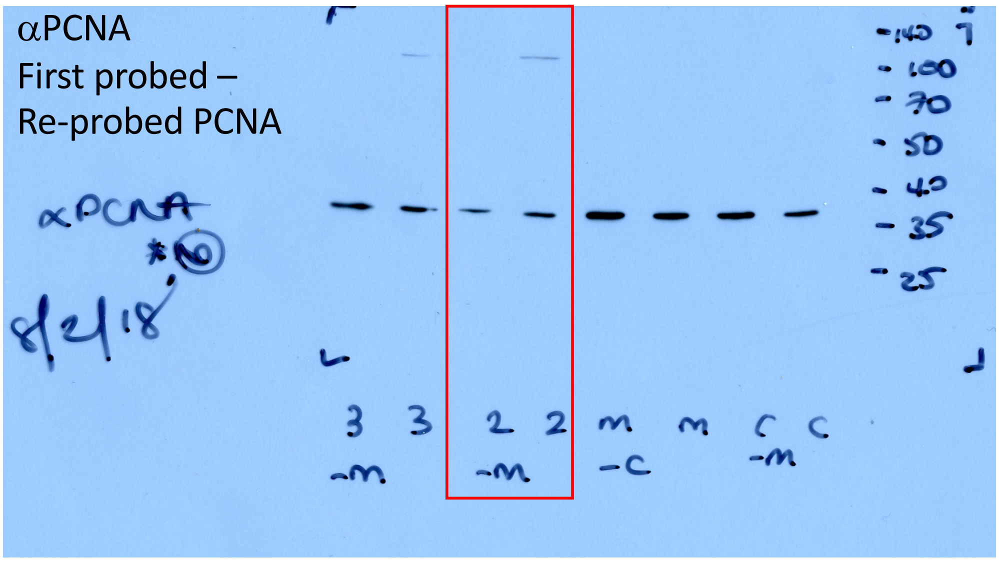

Extended data figure 1d continued

IP/Western blots probed with antibodies as indicated.

MTA2 Pull down / Probed αCHD4, αMTA2, αGATAD2A

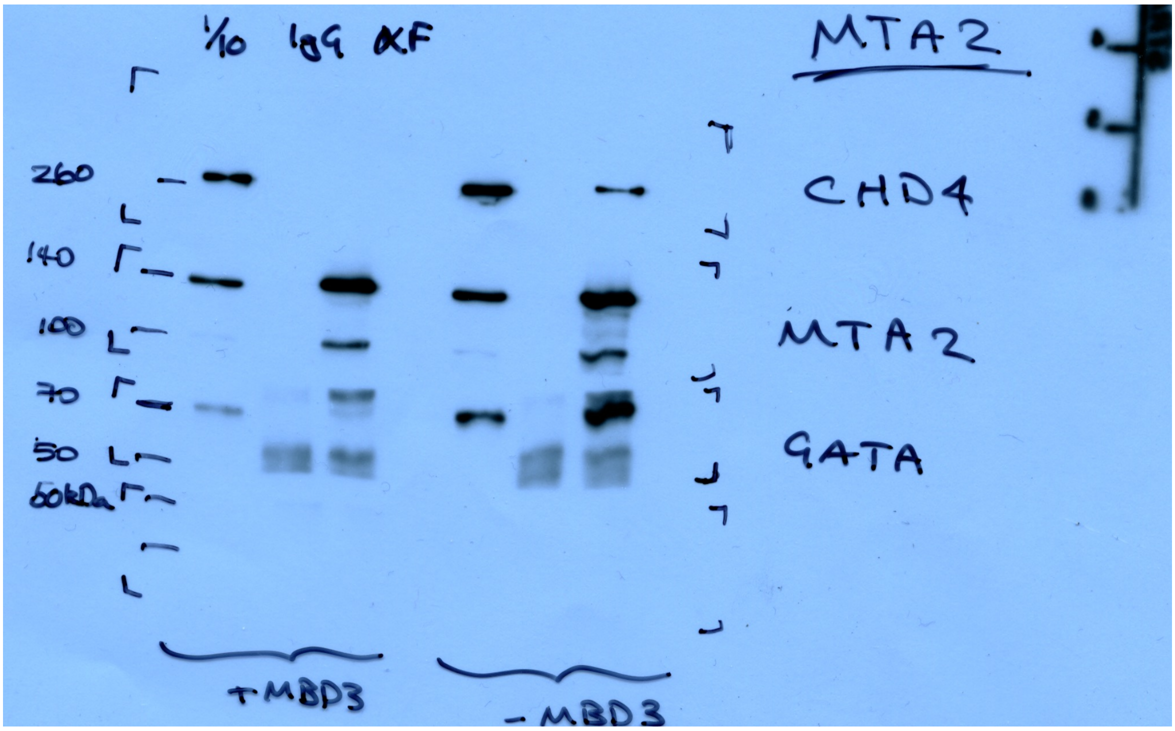

MTA2 Pull down / Probed αMBD3

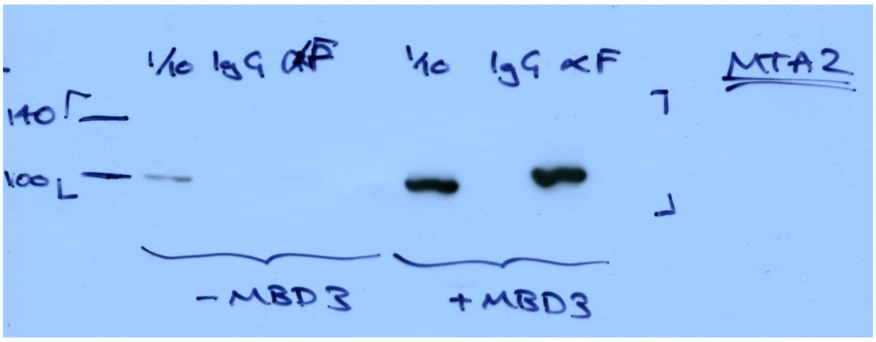

Supplement: Supplementary file 16 — Unprocessed blots. [file 41594_2023_1095_MOESM16_ESM.pdf]
