# Supplementary figures and images for: Live-cell three-dimensional single-molecule tracking reveals modulation of enhancer dynamics by NuRD
Source: Nat Struct Mol Biol. 2023 Sep 28;30(11):1628–39. doi: 10.1038/s41594-023-01095-4 (PMC10643137; doi:10.1038/s41594-023-01095-4)

Basu et al.

**Extended Data Figure 3b**

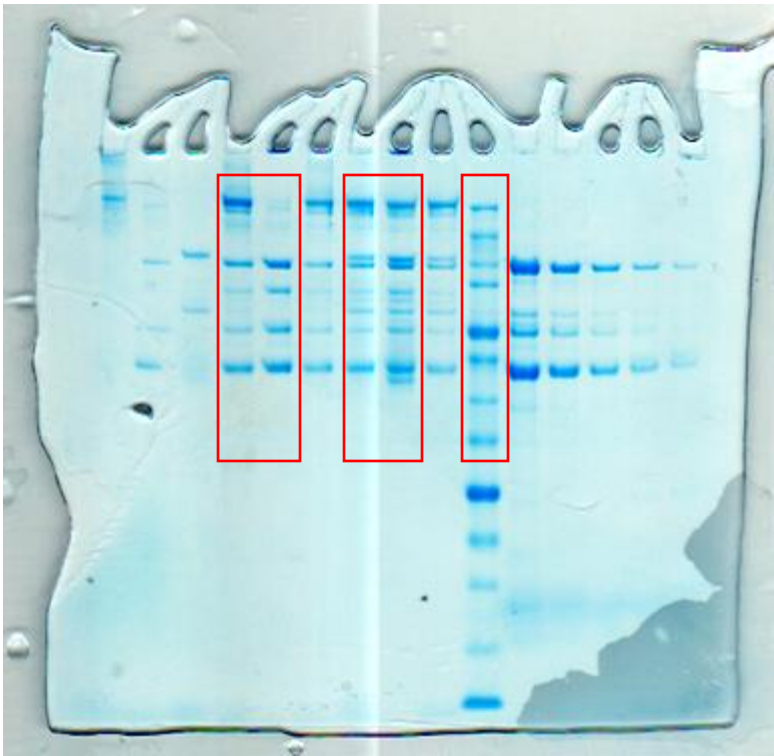

**Extended Data Figure 4a**

CHD4+DNA

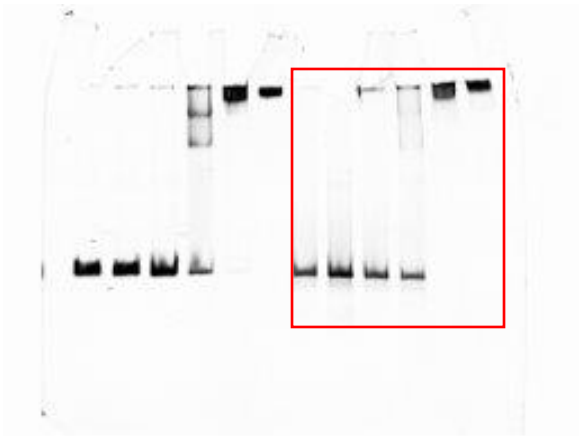

CHD4+Nuc

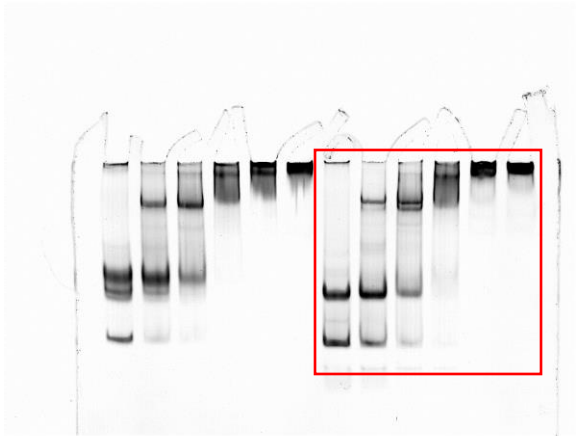

GATAD2A+Nuc

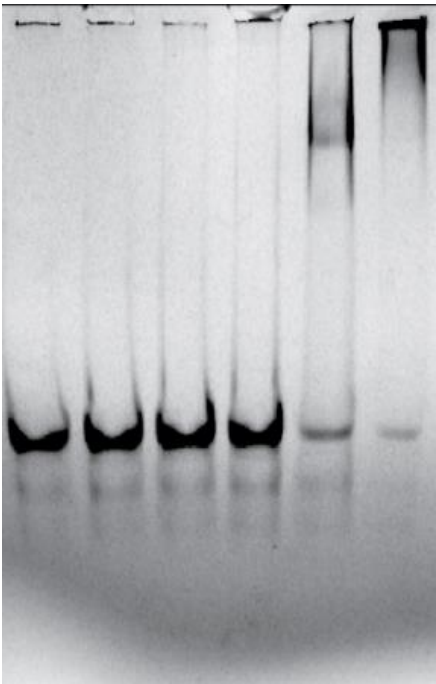

PMMR+Nuc

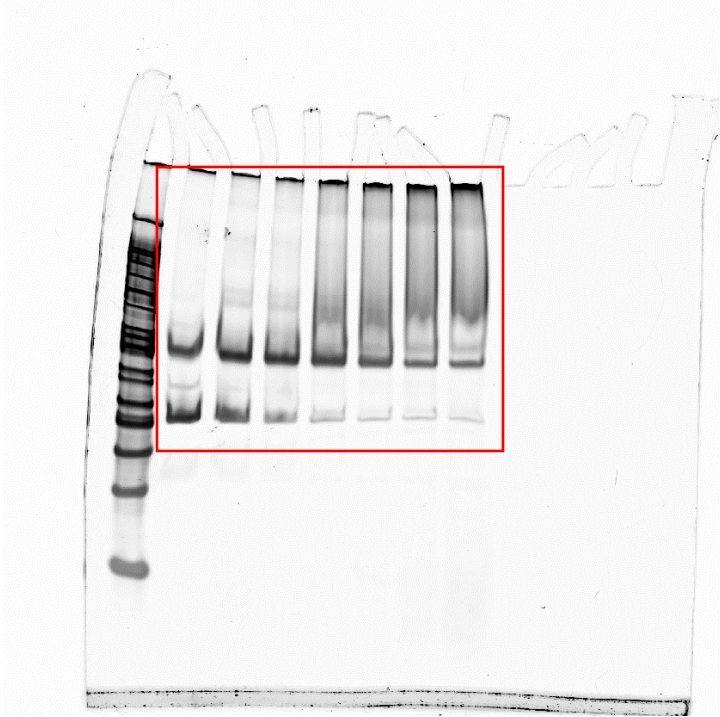

Supplement: Supplementary file 17 — Unprocessed blots. [file 41594_2023_1095_MOESM17_ESM.pdf]
